# Latent cytomegalovirus disrupts innate NK cell responses to *P. falciparum* and impairs parasite control in first infection in adults

**DOI:** 10.1371/journal.ppat.1014372

**Published:** 2026-06-23

**Authors:** Reena Mukhiya, Jessica R. Loughland, Nicholas L. Dooley, Zuleima Pava, Damian A. Oyong, Dean W. Andrew, Julianne Hamelink, Kiana Berry, James S. McCarthy, Bridget E. Barber, J. Alejandro Lopez, Christian R. Engwerda, Michelle J. Boyle

**Affiliations:** 1 Burnet Institute, Melbourne, Victoria, Australia; 2 QIMR Berghofer Medical Research Institute, Herston, Queensland, Australia; 3 Faculty of Medicine, Dentistry and Health Sciences, University of Melbourne, Carlton, Victoria, Australia; 4 School of Environment Sciences, Griffith University, Brisbane, Queensland, Australia; 5 School of Translational Medicine, Monash University, Clayton, Victoria, Australia; University of Iowa, UNITED STATES OF AMERICA

## Abstract

NK cells are innate and adaptive responders to malaria, with functional responses underpinned by NK cell heterogeneity. One driver of NK cell heterogeneity is latent CMV infection. Latent CMV infection negatively impacts adaptive immunity to malaria, but whether CMV-mediated changes to the NK cell compartment also impact innate responses to malaria is unknown. We investigated the impact of latent CMV infection on innate NK cell responses to the malaria parasite *in vitro* in malaria naïve adults, and *in vivo* NK cell responses during a first controlled human malaria infection. We found that transcriptional activation of NK cells by parasites was attenuated in CMV seropositive individuals. Further, during a first malaria infection, markers of NK cell activation and cytotoxicity were reduced. This attenuated response was not restricted to a single NK phenotype but occurred across diverse NK cell phenotypes. Consistent with a global NK cell attenuation, IL12 production from myeloid cells, a response that supports NK cell activation on exposure to *P. falciparum* parasites, was lower in CMV infected individuals. Linking NK cell activation to clinical outcomes, NK cell perforin expression was associated with parasite control in CMV seronegative individuals during first malaria infection. Data highlight the interplay between pathogens and the host-immune response that influence clinical outcomes.

## Author summary

How an individual’s immune system responds to infection is diverse, with multiple factors influencing the disease outcome. One such factor is individuals’ prior exposure to other pathogens. Cytomegalovirus (CMV) is a known driver of immune system diversity, which we have previously shown can modify the adaptive immune response to *Plasmodium falciparum* malaria. Here we used human cell samples from healthy individuals, and adults during controlled human malaria infection to study the impact of CMV on the innate immune response to malaria. Focusing in on NK cells, we show that latent CMV infection dampens the NK cell response to malaria in health individuals and during a first malaria infection in adults. This dampened NK cell response was seen across multiple NK cell types. Additionally, latent CMV infection was associated with reduced production of IL12 from myeloid cells. IL12 supports NK cell activation by malaria parasites. Of note, during a first malaria infection in adults, individuals with latent CMV infection had higher parasite growth rates, but lower clinical symptoms. Together data highlight the complex interplay between pathogens that can impact the immune response to infection.

## Introduction

*Plasmodium falciparum* malaria remains a significant global health challenge, with an estimated 282 million cases and over 600 000 deaths in 2024 [1]. Protective immunity to malaria is mediated by both innate and adaptive mechanisms that control parasites and limit immunopathology [2]. Natural Killer (NK) cells are key immune cells in both innate and adaptive responses to malaria and have multiple roles in protection [3]. NK cells are phenotypically heterogeneous, with this diversity and corresponding functionality influenced by multiple host and environmental factors [4,5]. One major driver of NK cell diversity is cytomegalovirus (CMV) infection which significantly modulates the NK cell compartment, driving expansion of ‘adaptive’ NK cells with hallmarks of memory including clonal expansion and modified responses to secondary challenge, including by heterologous stimulation [6–8]. We have recently shown that latent CMV infection is associated with reduced antibody development in malaria, both during a first infection and following vaccination [9]. However, whether latent CMV infection can also impact innate NK cell responses to malaria is unknown.

Human NK cells are defined based on CD16 and CD56 expression as CD16^-^CD56^bright^, CD16^+^ CD56^dim^ and CD16^+^CD56^neg^ cells and they express a vast array of activating and inhibiting receptors [4,10–12]. Recent studies have highlighted the key role of NK cells in malaria immunity, identifying NK cell receptors which can directly recognise *P. falciparum* parasites, and parasite-mediated NK cell inhibitor mechanisms [13,14]. In response to parasites, NK cells responded innately, rapidly producing IFNγ, a key cytokine for parasite control, along with cytotoxic molecules, a process that also requires NK cell activating cytokines IL12 and IL18 from myeloid cells [15–18]. In mouse models, innate NK cell responses have been directly linked to parasite control and protection in some studies [19,20]. NK cells also have roles as effectors of antibody dependent cellular cytotoxicity (ADCC) targeting malaria, via adaptive immune mechanisms which are associated with protection in children [12,21,22]. The ability of NK cells to respond to malaria, through both innate and ADCC pathways, is heterogeneous in both malaria naïve and malaria exposed subjects [15,16,23,24]. This heterogeneity is in part mediated by underlying diversity in expression of NK cell receptors. For example, for innate NK cell responses, a subpopulation of NK cells expressing the lectin-type receptor NKG2A is the predominant NK phenotype producing IFNγ in response to *P. falciparum* parasites *in vitro* [15]. [12,21]

One of the most significant drivers of heterogeneity of the NK cell compartment is CMV infection [6]. CMV is a ubiquitous beta-herpes virus which establishes an asymptomatic, life-long infection and has a global prevalence ranging from ~50% in high income countries, to up to 100% in some areas of Africa where it is acquired early in life [25]. Latent CMV drives expansion of adaptive CD56^dim^ CD57^+^ NKG2C^+^ NK cells which are characterized by reduced IFNγ and cytotoxic responses to exogenous IL12 and IL18 [6,26]. These CMV expanded NK cells, express lower frequencies of the Natural Cytotoxic Receptors (NCRs; for example, NKp30 and NKp46), and higher frequencies of inhibitory Killer-cell immunoglobulin-like receptors (KIRs) such as NKG2D [26–29]. These changes have important consequences for responsiveness of NK cells to other pathogens and stimuli. For example, both IFNγ production and degranulation are reduced in CD57^+^NKG2C^+^ NK cells when Peripheral Blood Mononuclear Cells (PBMCs) from CMV seropositive UK adults are stimulated with pertussis or H1N1 influenza vaccine antigens [8]. Here we investigate the impact of CMV on the innate NK cell responsive to *P. falciparum* parasites in malaria naïve individuals *in vitro* and explore the consequences of changes to parasite control in first infection. Data highlight the complex interplay between individuals lived histories to infecting pathogens and the host-immune response during infection.

## Results

### The innate transcriptional response of NK cells to *P. falciparum* is modulated by latent CMV infection

To dissect the impact of latent CMV infection on innate NK cell responses to malaria, we used two approaches 1) analysis of responses *in vitro* in malaria naïve, healthy donors, and 2) analysis of responses *ex vivo* from participants in controlled human malaria infection studies, using a subset of samples available from previous cohorts [9,30]. CMV serostatus was determined by plasma antibodies to CMV via ELISA, and innate responses compared between CMV seronegative and seropositive individuals (S1 Table).

**Fig 1:**
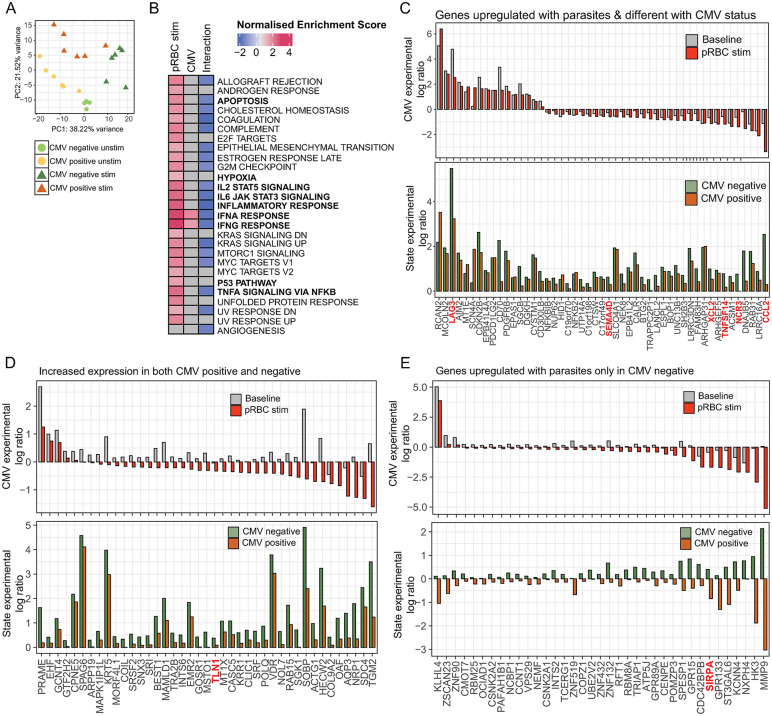
Transcriptional profiling of NK cells after stimulation with P. falciparum in CMV seronegative and seropositive individuals. **(A)** NK cells from CMV seronegative (n = 6) and CMV seropositive (n = 6) individuals were analysed by RNAseq *ex vivo* and following stimulation with *P. falciparum* infected RBC (pRBC). Principal component analysis (PCA) of DEGs identified by glmmSeq, with individuals’ CMV and stimulation status indicated. **(B)** GSEA pathway analysis of all DEGs identified as significantly modulated by parasite stimulation (pRBC), CMV serostatus, and either both or an interaction term. Normalised enrichment score for hallmark pathways shown. **(C)** DEGs that were significant for CMV status and were upregulated following stimulation, but without interaction term. **(D/E)** DEGs that had a significant interaction term between CMV serostatus and stimulation, which were upregulated in both CMV seronegative and CMV seropositive **(D)**, or only upregulated in CMV seronegative individuals (**E)**. For (**C-E)** Top panels are the CMV serostatus log ratio at baseline (grey) or following stimulation (red). A positive log ratio indicates genes which were significantly higher in CMV positive individuals. Bottom panels are State log ratio in CMV seronegative (green) and CMV seropositive (orange) individuals. A positive log ratio indicates genes that were upregulated following stimulation with pRBCs. See also S1 Fig and S2 Table.

First, to assess if CMV serostatus has the potential to modify the response to malaria parasites, we assessed the transcriptional profile of NK cells following *in vitro* stimulation with *P. falciparum* parasite infected red blood cells (pRBCs) from CMV seronegative and CMV seropositive healthy, malaria naïve individuals (S2 Table). PBMCs were cultured for 24 hours with parasites or media, and then NK cells isolated with negative bead selection for sequencing (CMV seronegative n = 6, age range 29–36, 50% male and CMV seropositive n = 6, age range 26–45, 50% male) (S1A, S1B Fig). NK cells from CMV seronegative and CMV seropositive donors were transcriptionally distinct prior to and following stimulation (Fig 1A). Differentially expressed genes (DEGs) with CMV serostatus (negative compared to positive), parasite stimulation (unstimulated compared to stimulated), and genes which responded in a CMV specific manner (significant for CMV serostatus and stimulation, and/or with a significant interaction term between CMV and stimulation) were identified using *glmmSeq* [31], which fits a negative binomial mixed-effects model at the individual gene level. Using this approach, 323 DEGs were identified that were modulated by CMV serostatus, consistent with the reported modulations of NK cells in CMV infection [26,32,33] (S1C Fig, S2 Table). In unstimulated cells, DEGs upregulated in CMV seropositive individuals included known markers of CMV-mediated NK cell changes, including *B3GAT1 (*encoding CD57) [32], and *LILRB1* [34] (S1E Fig). While *KLRC2* (encoding NKG2C) was not identified, this gene was significantly higher in CMV seropositive individuals in unadjusted analysis (p = 0.004, q (adjusted)=0.11) (S2 Table).

In response to parasite stimulation, 9578 DEGs were identified, consistent with a robust response of NK cells to parasites (S1C Fig, S2 Table). Of these genes, 259 (2.7%) also differed significantly by CMV serostatus (indicating that the expression differed by CMV status before and after stimulation, 148 DEGs) and/or had a significant interaction with stimulation (indicating that the response to parasites were different based on CMV serostatus, 111 DEGs). Gene set enrichment analysis (GSEA) of DEGs identified upregulation of major inflammatory pathways including in response to parasite stimulation (Fig 1B). However, for DEGs modified by CMV only IFNA and IFNG response pathways were identified. For DEGs that were identified for both parasite stimulation and CMV serostatus, or had an interaction term, GSEA showed down regulation of hallmark signatures including inflammatory pathways that were otherwise upregulated in response to parasites, suggesting that NK cells from CMV seropositive individuals had an attenuated response to parasite stimulation. Indeed, analysis of individual DEGs that were identified for both parasite stimulation and CMV, or had a significant interaction term, suggested important functional differences (Fig 1C–1E). For example, genes upregulated in response to parasites included *LAG3,* expression of which was higher in CMV seropositive individuals both before and after stimulation (Fig 1C). The inhibitory receptor LAG3 has recently been shown to inhibit NK cell IFNγ production and proliferation both in healthy and HIV infected individuals [35]. In contrast, genes *CCL2* (which is essential for monocyte recruitment) and *NCR3 (*encoding the NK cell activating receptor NKp30) were higher in CMV seronegative individuals, both before and after parasite simulation (Fig 1C). Furthermore, *SEMA4D* (CD100), reported to enhance NK cell killing [36]; *TNFSF14,* which has been shown to increase with NK cell activation and induce DC maturation [37]; and *XCL2,* a key NK cell chemoattractant that recruits DCs [38], were all higher in CMV seronegative individuals. Of DEGs with a significant interaction term between stimulation and CMV serostatus (indicating a significantly different response to parasites between the groups), 41 DEGs were significantly less activated in CMV seropositive individuals and/or significantly lower in CMV seropositive individuals following parasite stimulation, and a further 36 DEGs were only upregulated in CMV seronegative individuals (Fig 1D, 1E). Amongst these DEGs were *TLN1* (encoding talin-1), essential for NK cell cytotoxicity by mediating adhesion and cell polarization [39], and *SIRPA* (encoding SIPRα), which is upregulated in NK cells after cytokine activation [40]. Taken together these DEGs suggest decreased NK cell responses to *P. falciparum* parasite stimulation in CMV seropositive individuals.

### Innate NK cell activation during a first malaria infection is reduced in CMV seropositive individuals

To assess if CMV serostatus modulated innate NK cell activation to *P. falciparum* parasites *in vivo,* we analysed responses in individuals during controlled human malaria infection (CHMI) (CMV seronegative n = 9, 26 years median (IQR 20–31), male 90%, CMV seropositive n = 11, 25 years median (IQR 21–29), male 72.7%, S1 Table). NK cells were analysed based on CD56 expression to identify CD56^bright^, CD56^dim^ and CD56^neg^ subsets (S2A Fig). Overall, there were limited differences between CMV seronegative and seropositive donors for total frequencies of NK cells. Further, the proportions CD56^bright^, CD56^dim^ cells were similar before and during CHMI (S2B Fig). Following *P. falciparum* infection, there was a significant increase in CD56^bright^ NK cells and a decrease in CD56^neg^ NK cells in both CMV seronegative and CMV seropositive individuals (S2B-S2C Fig).

**Fig 2:**
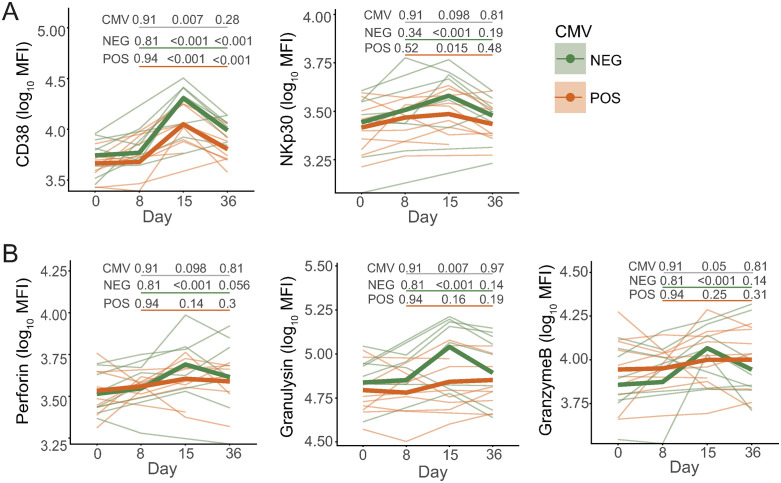
CD56^dim^ NK cell activation during controlled human malaria infection in CMV seronegative and seropositive individuals. CD56^dim^ NK cell activation was analysed by quantifying median fluorescence intensity (MFI) of **(A)** activation markers CD38 and NKp30, and **(B)** cytotoxic potential quantified by markers Perforin, Granzyme B and Granulysin in CMV seronegative (n = 9) and CMV seropositive individuals (n = 11). Data are log_10_ MFIs of markers with thin lines representing individual data coloured by CMV serostatus, and bold lines representing the mean of the predicted values from the fitted models for each group. P values are from linear mixed effect models. CMV is p values for the interaction term between each timepoint (compared to day 0) and CMV serostatus (underlined in grey). NEG/POS are P values for the comparison between day 0 and each subsequent timepoint for CMV seronegative individuals (NEG, underlined in green) and CMV seropositive individuals (POS, underlined in orange) which were determined from contrasts. See also Fig 2.

Innate NK cell activation and cytotoxic potential during infection was quantified as the median signal intensity of CD38, NKp30, Perforin, Granulysin and GranyzmeB on each NK subset at day 0, 8, 15 and 36, and analysed with linear mixed effects models (S2A Fig). For CD56^dim^ NK cells, expression of activation markers CD38 and NKp30 significantly increased in both CMV seronegative and seropositive individuals, with the highest expression at day 15 post infection (Fig 2A). However, the magnitude of the increase for CD38 was significantly higher in CMV seronegative individuals (mixed model interaction term p = 0.007) (Fig 2A). For cytotoxic markers Perforin, Granulysin and GranyzmeB, increased expression was only detected in CMV seronegative individuals following CHMI (Fig 2B). Additionally, for CD56^bright^ cells, while all activation and cytotoxic markers increased in CMV seronegative and seropositive individuals during malaria, this increase was significantly higher in CMV seronegative individuals (S2D Fig). Similarly, for CD56^neg^ NK cells, a relatively increased response for CD38, Granulysin and Granzyme B was also observed in CMV seronegative individuals (S2E Fig). Together these data suggest that CMV infection has a negative impact on malaria-induced activation of CD56^bright^, CD56^dim^ and CD56^neg^ NK cells during a primary malaria infection in adults, consistent with *in vitro* transcriptional findings.

### CMV associated changes to the NK cell compartment are maintained during CHMI

**Fig 3:**
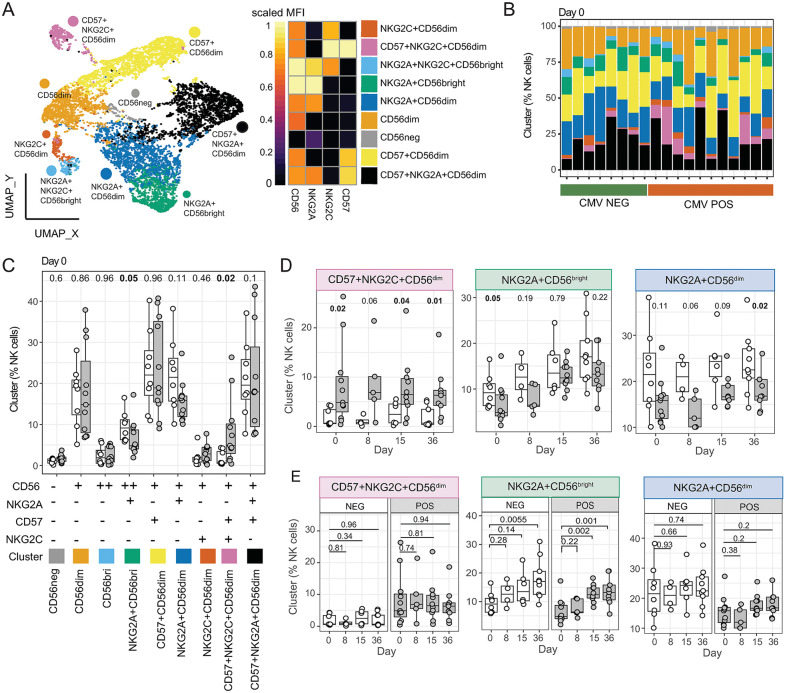
Distribution of NK cell phenotypes in CMV seronegative and seropositive individuals during CHMI. **(A)** NK cell phenotypes were classified based on expression of CD57, NKG2A, NKG2C and CD56. UMAP projection of NK cell clusters, and heatmap of cluster maker expression on each cluster. **(B)** Percentage of each NK cell cluster in CMV seronegative (n = 8) and CMV seropositive (n = 9) individuals at day 0, prior to malaria inoculation. **C)** Proportion of NK cell subsets between CMV seronegative and CMV seropositive individuals. Marker expression of each cluster is indicated. **(D/E)** CD57 + NKG2C+CD56^dim^, NKG2A+CD56^bright^ and NKG2A+CD56^dim^ cells comparing CMV seronegative and CMV seropositive individuals at each CHMI timepoint **(D)** and comparing day 0 to each subsequent timepoint (**E)**. For **(C-E)** data are Tukey boxplots with the median, 25^th^ and 75^th^ percentiles. The upper and lower hinges extend to the largest and smallest values, respectively but not further than 1.5X IQR from the hinge. Individual data are shown as points. For **C/D** p is Mann-Whitney U test. For **E** p is Wilcoxon signed-rank test.

NK cell phenotypes are modulated by CMV, which induces expansion of CD56^dim^ CD57^+^/NKG2C^+^ NK cells, resulting in a proportional decrease of NKG2A^+^ NK cells within CMV seropositive individuals [6]. Because NKG2A^+^ NK cells have been reported to preferentially respond to *P. falciparum in vitro* [15], we hypothesised that reduced activation of NK cells during malaria may be due to reduced frequencies of this phenotype. To investigate this hypothesis, NK cell phenotypes were analysed based on expression of NKG2A, NKG2C, CD57, together with CD56, in the same individuals analysed in Fig 2 before, during and after CHMI. Clustering of NK cells based on these markers identified nine NK cell clusters which included two CD56^bright^ subsets, one co-expressing NKG2A, and the other expressing both NKG2A and NKG2C; six CD56^dim^ subsets, including a CD57^+^NKG2C^+^cluster, and a NKG2A^+^ cluster; and a small cluster of CD56^neg^ cells (Fig 3A). Prior to malaria infection, distribution of NK clusters was heterogenous at the individual level (Fig 3B). However, consistent with prior studies, there was a significantly increased proportion of CD57^+^NKG2C^+^CD56^dim^ NK cells (p = 0.02), and a decreased proportion of NKG2A^+^CD56^bright^ (p = 0.05) and NKG2A^+^CD56^dim^ (p = 0.11) cells in CMV seropositive individuals prior to malaria (Fig 3C). For CD57^+^NKG2C^+^CD56^dim^ cells, these CMV-associated differences were maintained over the course of infection (Fig 3D,3E). For NKG2A^+^ cells, CMV-associated differences were only maintained for NKG2A^+^CD56^dim^ and not NKG2A^+^CD56^bright^ cells, the latter of which expanded in both CMV seronegative and CMV seropositive individuals during CHMI (Fig 3D,3E).

### Expression of activation and cytotoxic potential markers is lower in CMV seropositive individuals during a first malaria infection across multiple NK cell phenotypes

**Fig 4:**
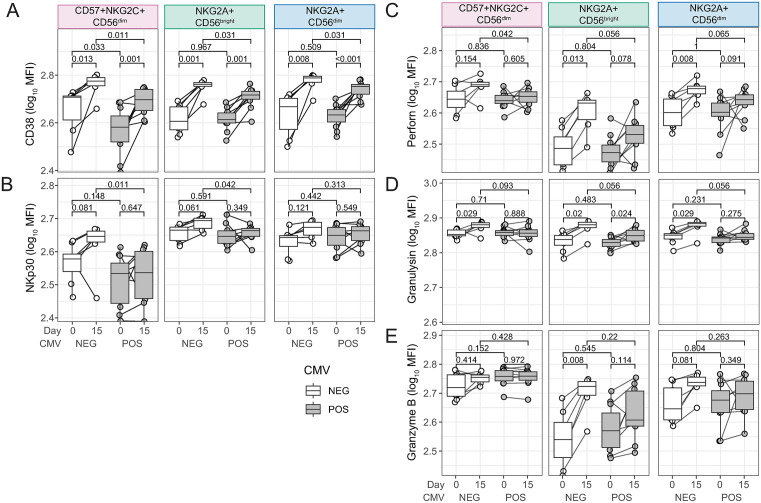
Activation and cytotoxicity in CMV modulated phenotypes during CHMI. Markers of activation (**A** - CD38 and **B** - NKp30) and cytotoxic potential (**C** - Perforin, **D** - Granulysin, **E** – Granzyme B) were quantified (median fluorescence intensity, MFI) in NK cells that were modulated by CMV infection, CD57 + NKG2C+CD56^dim^, NKG2A+CD56^bright^ and NKG2A+CD56^dim^ phenotypes. Expression was compared between day 0 and day 15 in CMV seronegative (n = 8) and CMV seropositive individuals (n = 9), and expression at day 0 or at day 15 was compared between groups. Data are Tukey boxplots with the median, 25^th^ and 75^th^ percentiles. The upper and lower hinges extend to the largest and smallest values, respectively but not further than 1.5X IQR from the hinge. Individual data are shown as points. For comparisons between groups P is Mann-Whitney U test. For comparisons within groups between days P is Wilcoxon signed-rank test. See also S3 Fig.

To assess if CMV-associated changes to the NK cell compartment underpinned the impaired NK cell responsiveness during malaria, we analysed markers of activation (CD38 and NKp30) and cytotoxicity potential (Perforin, Granulysin, and Granzyme B) across all identified phenotypes in the same individuals during CHMI. We focused on day 15 after CHMI which was the peak of NK cell activation (Fig 2) and first analysed NK cells that were influenced by CMV serostatus (Fig 3). Robust activation indicated by increased expression of CD38 was detected in CD57^+^NKG2C^+^CD56^dim^, and NKG2A^+^CD56^bright^ and NKG2A^+^CD56^dim^ cells at day 15 in both CMV seronegative and CMV seropositive individuals (Fig 4A). However, at day 15, CD38 expression was higher in CMV seronegative individuals for all phenotypes (Fig 4A). CHMI mediated increases of NKp30 were less robust and did not reach statistical significance in either CMV seronegative or CMV seropositive individuals in any NK cell phenotype analysed (Fig 4B). For markers of cytotoxic potential, there was also evidence for stronger activation at day 15 of CHMI in CMV seronegative individuals across phenotypes, with significantly increased Perforin expression in both NKG2A^+^ expressing cells (Fig 4C), increased Granulysin expression across all phenotypes (Fig 4D), and increased Granzyme B expression in NKG2A^+^CD56^bright^ cells (Fig 4E). In contrast, for CMV seropositive individuals, malaria only drove increased cytotoxic markers for Granulysin in NKG2A^+^CD56^bright^ cells (Fig 4C-4E). This pattern of more robust activation and larger increases in cytotoxic markers in CMV seronegative subjects was seen in all other NK cell phenotypes (S3A-S3E Fig). For CD38, while expression increased at day 15 for most phenotypes (except CD56^neg^ cells) regardless of CMV infection status, expression was significantly higher in CMV seronegative individuals (S3A Fig). Further, for NKp30, there was increased expression at day 15 after CHMI in 4/6 additional NK cell phenotypes, but no induction detected in CMV seropositive individuals (S3B Fig). Similarly, Perforin expression increased in 5/6 phenotypes in CMV seronegative individuals and only in 1/6 for CMV seropositive; Granulysin increased in 4/6 phenotypes in CMV seronegative subjects but did not increase in any CMV seropositive individuals (S3C-S3D Fig). For Granzyme B, responses were more varied; increased expression only reached statistical significance for a single phenotype amongst the CMV seropositive individuals (S3E Fig). Taken together, these data indicate that CHMI induces robust activation across multiple NK cell phenotypes, and that increases in markers of cytotoxic potential are consistently more robust in CMV seronegative individuals.

### Monocyte IL12 production in response to TLR4 stimulation is higher in CMV negative individuals

**Fig 5:**
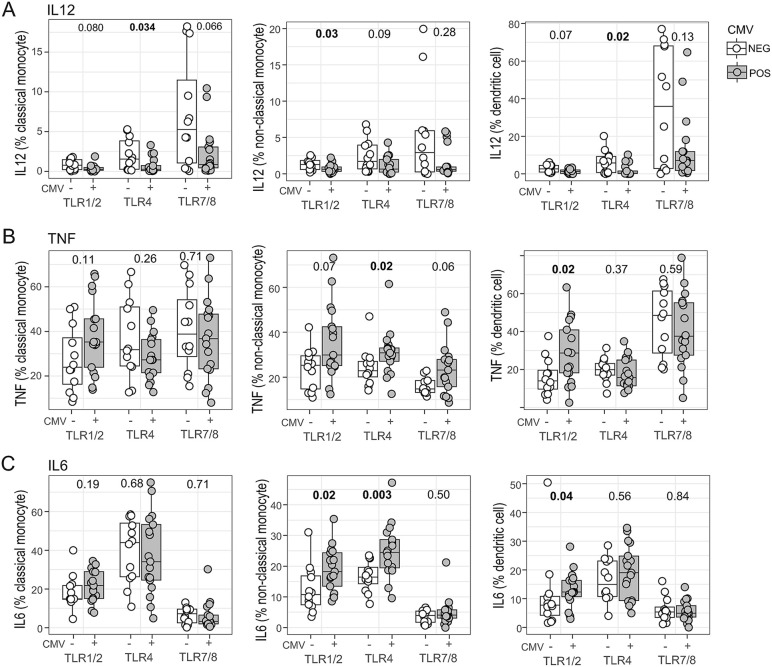
Cytokine production following TLR stimulation in myeloid cells in CMV seronegative and seropositive individuals. Whole blood from healthy CMV seronegative (n = 12) and CMV seropositive (n = 17) donors was stimulated with TLR agonists Pam3Csk4/HKLM for TLR1/2, LPS for TLR4 and CL075 for TLR7/8 and IL12 **(A**), TNF **(B)** and IL6 **(C)** quantified in classical monocytes, non-classical monocytes and dendritic cells. See also S4 Fig.

In previous studies, it has been shown that together with parasite contact, the cytokines IL12 and IL18 are essential for NK cell activation *in vitro* by *P. falciparum* [15–18]. Because our data suggested that NK cell activation and cytotoxic potential was disrupted across diverse NK phenotypes during a first malaria infection in CMV seropositive individuals, we hypothesized that CMV infection may reduce the cytokine milieu from myeloid cells that supports NK cell activation. To test this hypothesis, we stimulated whole blood from additional healthy, malaria naïve donors with innate cell agonists Pam3Csk4/HKLM, LPS and CL075, which activate via TLR1/2, TLR4 and TLR7/8 respectively, (CMV seronegative n = 12, age range 18–53 years, male 50%, CMV seropositive n = 17, age range 19–55 years, male 58.8%, S1 Table). These TLR pathways have been shown to be activated by *P. falciparum* including: TLR2 and TLR4 recognition of Glycosylphosphatidylinositol (GPI) anchors [41], TLR4 binding hemozoin bound with parasite proteins [42], and TLR8 responding to *Plasmodium* RNA [43]. Following stimulation, IL12, TNF and IL6 were quantified by intracellular staining in classical and non-classical monocytes and dendritic cells (DCs) (S4A, S4B Fig). In both classical monocytes and dendritic cells, significantly lower IL12 production was measured in response to TLR4 stimulation in CMV seropositive individuals (Fig 5A). In non-classical monocytes, IL12 production was significantly reduced in CMV seropositive individuals in response to TLR1/2 stimulation (Fig 5A). In contrast, TNF production was higher in CMV seropositive individuals after TLR4 stimulation in non-classical monocytes and TLR1/2 stimulation in dendritic cells (Fig 5B). Additionally, levels of IL6 were higher in CMV seropositive individuals after TLR1/2 and TLR4 stimulation in non-classical monocytes, and TLR1/2 stimulation for dendritic cells (Fig 5C). Thus, data suggests that modified myeloid cell responses in CMV seropositive individuals may be a contributing factor in modulated NK cell responses to malaria parasite *in vitro* and *in vivo* during a first malaria infection.

### Parasite multiplication rate is higher in CMV seropositive individuals and associated with NK cell activation

**Fig 6:**
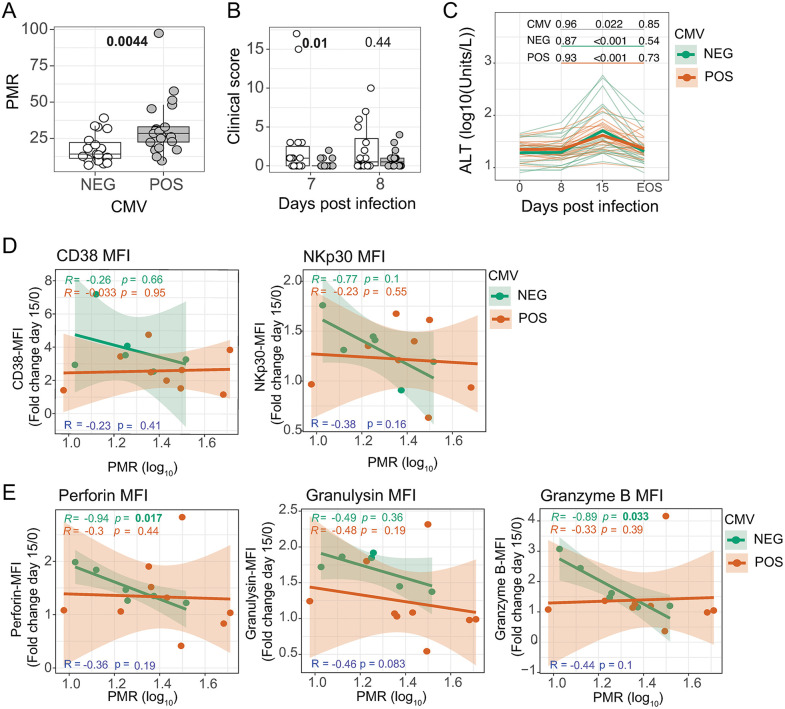
Parasite multiplication rate, clinical parameters and NK cell associations. Associations between CMV serostatus and parasite control and indicators of inflammation were investigated in a cohort of individuals in CHMI (CMV seronegative n = 19, CMV seropositive n = 21). **A**) Parasite multiplication rate (PMR) was compared between CMV seronegative and seropositive individuals. **B)** Clinical score prior to treatment between CMV seropositive and seronegative. For **A-B** data are Tukey boxplots with the median, 25^th^ and 75^th^ percentiles. The upper and lower hinges extend to the largest and smallest values, respectively but not further than 1.5X IQR from the hinge. Individual data are shown as points. For comparisons between groups P is Mann-Whitney U test. For **A/B** P is Mann Whitney U test. **C)** Alanine transaminase at day 0, 8, 15, EOS. Data are log_10_ ALT with thin lines representing individual data coloured by CMV serostatus, and bold lines representing the mean of the predicted values from the fitted models for each group. P values are from linear mixed effect models. CMV is P values for the interaction term between each timepoint (compared to day 0) and CMV serostatus (underlined in grey). NEG/POS are P values for the comparison between day 0 and each subsequent timepoint for CMV seronegative individuals (NEG, underlined in green) and CMV seropositive individuals (POS, underlined in orange) which were determined from contrasts. **D/E)** Fold change increase in expression of activation markers CD38 and NKp30 **(D)**, or cytotoxic markers Perforin, Granulysin, or Granzyme B **(E)** on CD56^dim^ NK cells was calculated for day 15 during CHMI and correlated with parasite multiplication rate (PMR) in a subset of individuals. CMV seronegative individuals are green (n = 8) and CMV seropositive individuals are shown in orange (n = 9). Spearman’s Rho and P are shown for CMV seronegative (green), CMV seropositive (orange) or all individuals (purple).

To assess the clinical relevance of CMV-mediated changes to NK cells and other innate cell responses to malaria, we analysed associations between CMV serostatus and early parasite control and inflammation in 40 individuals during CHMI (S3 Table). This cohort included all individuals analysed for NK cell responses during CHMI (Figs 2-4), along with additional participants previously studied for the impact of CMV seropositivity on antibody induction [9,30]. Within this cohort, we recently reported that CMV is not associated with parasite burden observed over the total infection period (before and after treatment) [9]. However, total parasite burden is influenced by both parasite growth and drug efficacy, therefore we assessed the association between CMV and parasite multiplication rate (PMR) prior to treatment. PMR was significantly higher in CMV seropositive individuals (Fig 6A). In contrast, the combined malaria clinical score, an indicator of inflammation [44], was significantly lower in CMV seropositive individuals, particularly early in infection (Fig 6B). Consistent with this, the malaria induced increase of alanine transaminase (ALT), a marker of tissue damage, was significantly greater in CMV seronegative individuals (Fig 6C). To link these CMV-associated changes to parasite control to reduced activation of NK cells in CMV seropositive subjects, we assessed the association between expression of activation and cytotoxic potential markers on CD56^dim^ NK cells and PMR. There was no association between the magnitude of activation (fold increase of CD38 or NKp30 at day 15 compared to baseline) and PMR in either CMV seronegative, CMV seropositive, nor total individuals (Fig 6D). However, there was a strong and significant negative correlation between the increase in cytotoxic potential markers Perforin and Granzyme B and PMR in CMV seronegative individuals (Fig 6E). This relationship was not seen in CMV seropositive individuals, or when CMV serostatus was not considered. Together, these data indicate CMV infection negatively reduces the capacity of NK cells to respond to *P. falciparum* parasites, reducing parasite control and malaria symptoms.

## Discussion

NK cells have key roles in protective immunity, participating in both innate and adaptive responses to pathogens, including malaria. Here we show that the innate NK cell responses to malaria are significantly modified by latent CMV infection. CMV seropositive individuals have reduced transcriptional responsiveness and reduced IFNγ and TNF gene expression pathways following *P. falciparum* parasite stimulation *in vitro,* and reduced NK cell activation and cytotoxic potential *in vivo* during a first malaria *P. falciparum* infection. This attenuated responsiveness to parasites was seen in CD56^bright^, CD56^dim^ and CD56^neg^ NK cells of diverse phenotypes and was not limited to NK cells expanded by CMV infection, suggestive of a systemic impact of CMV, possibly linked to myeloid cell changes. Of clinical relevance, the PMR was greater in CMV seropositive individuals during a first malaria infection, which was associated with reduced expression of markers associated with NK cell cytotoxic potential. Together, data identify an important influence of CMV on innate NK cell function and response to *P. falciparum* infection and malaria, and highlight the complex interplay being individuals’ infection history and immune responses to pathogens.

Heterogeneity in human immune responses to pathogens is well recognised, and in malaria can range from low grade, controlled and asymptomatic, to high parasitemia and death. Underpinning these heterogeneous outcomes is the intersection of host and parasite factors, including the expression of parasite virulence genes [45,46], host genetics [47], age [48–51] and prior exposure and acquired immunity [52]. Our findings additionally highlight how an individual’s pathogen exposure history influences immune activation and disease outcome during a first malaria infection. Specifically, we show that latent CMV modulates the innate cell responses to *P. falciparum*, particularly within the NK cell compartment. It is well recognised that latent CMV infection drives the expansion of ‘adaptive’ CD56^dim^ NK cells, expressing both CD57 and NKG2C, and proportional decreases in NKG2A [6]. We also confirmed these CMV-associated changes within NK cells within our cohorts. However, we observed reduced expression of markers of activation and cytotoxic potential across all NK cell phenotypes studied, suggesting a globally attenuation of the innate NK cell response to malaria. Consistent with this, IL12 production induced by TLR1/2 or TLR4 from myeloid cells was reduced in CMV seropositive individuals; IL12 is a key supporting cytokine for NK cell activation in response to parasites. Thus, data suggest that the reduced innate NK cell response to *P. falciparum* in CMV seropositive individuals may be in part due to CMV-mediated changes to both NK cells and other cell compartments.

The potential role of CMV-mediated changes to myeloid cell compartments on innate cell activation during malaria is consistent with previous studies. Indeed, latent CMV drives changes across the immune landscape, with a systems analysis reporting that half of all measured immune parameters were impacted by CMV serostatus [53]. Here data add to these previous findings showing that cytokine production in response to different TLR agonists were modulated by CMV serostatus, and production of the NK-activating cytokine IL12 was lower in CMV seropositive individuals. *In vitro* studies have shown that CMV viral homology to IL10 (cmvIL10) can inhibit IL12 secretion induced by LPS (TLR4 stimulation) in DCs [54]. CmvIL10 also reduced IL6 and TNF production, in contrast to data presented here where these cytokines were elevated in CMV seropositive individuals. While CMV DNA is rarely detected in circulating cells [55,56], it has been detected in bone marrow CD34^+^ hemopoietic progenitor cells from healthy CMV seropositive individuals [57]. Analogous to innate cell training, it is possible that latent infection within the hemopoietic compartment imprints myeloid cells to alter their responsiveness to pattern recognition signals [58]. Thus, further studies are required to better understand how latent CMV infection within stem cells or elsewhere modulates the myeloid cell compartment and the impact of these changes to NK cell responsiveness to *P. falciparum* and other stimuli.

NK cells have key roles in protection from malaria, implicated as both innate cell responders and adaptative cells that control parasite growth via ADCC [2]. This study solidifies the importance of the innate NK cell response in early parasite control, identifying perforin expression, a potential marker of NK cell cytotoxicity, is associated with PMR during first *P. falciparum* infection in CMV seronegative individuals. Additionally, we have previously reported that latent CMV infection is associated with reduced antibody induction in the same CHMI study, highlighting that CMV has negative impacts on malaria immunity in both innate and adaptive responses [9]. Given that CMV is acquired very early in life in malaria endemic areas, our data highlight the importance of better understanding the interplay between infecting pathogens to understand the immune response to malaria in children living in malaria endemic areas. Indeed, while data here suggest that CMV has an immunomodulatory impact on host response to malaria, and thus may hamper parasite control, clinical symptoms were also reduced in CMV seropositive individuals. The impact of CMV on the severity of natural infection is unknown, and detangling these factors in natural infection in malaria endemic areas may not be currently possible given that CMV is almost universally acquired early in life [25]. However, vaccines against CMV are in development, including in Phase 3 studies [59], thus changes to CMV transmission via vaccination or other public health measures may have important implications to malaria endemic areas. Indeed, while reduced capacity to control parasites may indicate increased risk of hyper-parasitemia and severe disease, paradoxically, the reduced inflammatory response may be protective from immunopathogenesis. Indeed, while CHMI studies are powerful approaches to investigate immune responses during malaria in a controlled environment, CHMI participants are treated relatively early in infection and how our data translate to endemic areas and childhood infections remains to be investigated. Further, malaria infection itself also modifies the NK cell compartment, driving expansion of CD56^neg^ NK cells with increased ADCC capacity [12]. How such malaria induced changes interplay with the impact of CMV infection, as adaptive immunity to malaria develops, requires further investigation.

Limitations of our study are the relatively small number of NK cell receptors investigated, including those involved directly in parasite recognition [13,14]. Future studies that include more markers of NK cell diversity or unbiased approaches such as single cell RNA sequencing are required to comprehensively dissect how specific NK cell markers may influence responses to malaria and the impact of CMV infection. Further, while analysis leveraged *ex vivo* analysis of surrogate markers of cytotoxic potential of NK cells during CHMI, cytokine production (IFNγ or TNF), degranulation (quantified with CD107a), cytotoxicity and parasite killing was not directly assessed. Additionally, links between CMV, NK cells and parasite control were only investigated in malaria naïve adults in CHMI, and how results translate to children in endemic areas requires further investigation. Further, findings on the impacts on parasite control and clinical outcomes require replication in larger studies to confirm relevance across a range of cohorts. Due to cell sample limitations, we were unable to investigate NK cell responses in our complete CHMI cohort of 40 individuals and were unable to test NK cell responsiveness to parasite and/or cytokine stimulation *in vitro* in donors. Furthermore, we couldn’t directly link myeloid compartment changes to NK cell responses in CHMI within the same individuals, hampering our ability to directly link myeloid changes to NK cells. Further studies are required to understand the mechanistic links between CMV status, myeloid cell responses and innate NK cell compartments response to *P. falciparum* parasites. Due to high prevalence of EBV in our study cohorts, we were unable to investigate an additional role of this infection on NK cell responses and CMV mediated changes.

## Materials and methods

### Ethics statement

Written informed consent was obtained from all participants. Ethics approval for the use of human samples in the relevant studies was obtained from the Alfred Human Research and Ethics Committee for the Burnet Institute (#288/23 and #166/24), the Human Research and Ethics Committee of the QIMR-Berghofer Medical Research Institute (P1479).

### Study cohorts

Blood was collected from malaria naïve, healthy donors in Australia and from controlled human malaria infection studies (CHMI), and samples were selected based on availability for use across assays as outlined in S1 Table. CHMI studies were performed as previously described using the Induced Blood Stage Malaria (IBSM) model [9,30,60]. Individuals who were malaria naïve were inoculated by intravenous injection of 2,800 *P. falciparum* infected red blood cells and monitored for parasite growth with qPCR [61]. Blood samples were collected at baseline (day 0), peak-infection (day 8), day 14/15 (collectively called day 15) and at the end of study (27–36 days post infection, collectively indicated as day 36 or EOS). Samples were from four studies across six independent infection cohorts, completed between May 2015 and February 2017 [62–64]. Samples used here were collected from volunteers who consented to donate blood for immunological studies within the parent clinical trial and therefore a sample size estimation was not performed. PBMCs were isolated using Ficoll-Paque (Sigma, USA) density gradient centrifugation and cryopreserved in 10% DMSO/FBS. All available PBMCs from a cohort of 40 individuals previously reported were used for NK cell responses *ex vivo* [9]. PBMCs from healthy non-infected individuals were collected by the same processes for analysis of NK responses following *in vitro* stimulation with malaria parasites. For analysis of TLR responses in myeloid cells, whole blood was collected from malaria naïve healthy donors, prior to malaria infection from participants enrolled in CHMIs ACTRN12621000866808 [44] and ACTRN12620000995976 [65]. For all donors, CMV and EBV seroprevalence was assessed using plasma samples (at day 0 for all CHMI) by commercially available ELISA kits (ab108724 and ab108730), according to manufacturer’s instructions. EBV seroprevalence in our CHMI study was 87.5% [9], and consistently ~90% in heathy malaria naive donors, thus was not considered further in analysis. Clinical score at day 7 and 8 following inoculation was calculated by the number and severity of malaria-related symptoms.

#### Sex as a biological variable.

Both male and females were included in this study. For some parent CHMI studies, females of childbearing age were excluded and as such the sex is not evenly distributed.

### *P. falciparum* parasite culture

Packed red blood cells (RBCs) from donors were infected *in vitro* with the *P. falciparum* 3D7 parasite strain [66]. Packed RBCs for parasite culture were acquired from the Australian Red Cross. *P. falciparum-*infected RBCs (pRBCs) were cultured at 5% haematocrit in Roswell Park Memorial Institute 1640 media (RPMI) supplemented with AlbuMAX II (0.25%) and heat-inactivated human sera (5%). Cultures were incubated at 37 °C in 1% O_2_, 5% CO_2_, and 94% N_2_ gas mixture. Culture media was replaced daily, and parasite stage/parasitemia was monitored by Giemsa-stained blood smears. pRBCs were grown to 15% parasitemia and purified from uninfected RBCs (uRBCs) and early stage pRBCs via magnet separation to enrich mature trophozoite stage pRBCs. Purified pRBCs (>95% purity) were stored at -80 °C following addition of a Glycerolyte cryopreservant.

### Profiling NK cell transcriptional response to *P. falciparum* parasites

PBMCs were thawed in 10% FBS/RPMI and cultured 1:1 cell ratio with 1x10^6^ mature trophozoite stage pRBCs or media (unstimulated control) at 37^o^C, 5% CO2 in 96-well U-bottom plates for 24 hours. Following culture, NK cells were isolated using negative selection (MACS Miltenyi Biotec, kit#130-092-657) and cell purity and viability quantified by flow cytometry (S4 Table). Purity and viability were similar between CMV seronegative and seropositive, and unstimulated compared to parasite stimulated cells (S1A, S1B Fig). RNA was extracted from NK cells using the QIAGEN PicoPure RNA isolation kit (Applied Biosystems, KIT0204), and RNA quality confirmed with the 2200 TapeStation system (G2964AA) by High Sensitivity RNA ScreenTape (5067–5579). RNA sequencing libraries were constructed using the NEBNext Single Cell/Low Input RNA Library Prep Kit for Illumina (E6420S) and NEBNext Multiplex Oligos for Illumina (96 Unique Dual Index Primer Pairs) (E6440S). One sample from an unstimulated CMV negative female donor failed RNA extraction and was not sequenced. The libraries were sequenced using a paired-end NextSeq 500/550 high output kit v2.5 (150 cycles) (Cat #20024907).

### *Ex vivo* NK cell phenotyping during CHMI

PBMCs were thawed in 10% FBS/RPMI 1640 and 0.02% Benzonase, and 1x10^6^ cells were stained with antibody LAG3, Fc (1:100, BD Biosciences) and monoblock (1:20, Biolegend) at 37°C, 5% CO2 for 45 min. After two washes (1x PBS), PBMCs were stained with Viadye Red live dead for 15 mins at room temperature (RT), washed twice with 2% FBS/PBS. Then, PBMCs were pre-stained with TCR γδ at RT for 15 mins and then surface stained for 15 minutes at RT with fluorescent-tagged antibodies in 2% FBS/PBS (S5 Table). Following two washes with 2% FBS/PBS, cells were fixed and permeabilized with eBioscience Foxp3/Transcription Factor Staining Buffer for 20 mins on ice. Intracellular staining was performed for 30 mins on ice after 2 washes with 1x perm buffer (S5 Table) and PBMCs were fixed with BD Stabilizing Fixative (BD Biosciences) and resuspended in 1x PBS. Cells were acquired on the Cytek Aurora 5 laser cytometer within 24 hours.

### TLR whole blood stimulation and intracellular cytokine staining

To assess TLR responsiveness of innate cells 300 µl of whole blood was stimulated with Pam3Csk4/HKLM (TLR1, 0.1 µg/mL; TLR2, 10^8^ cells/mL), LPS (TLR4, 200 ng/mL) or CL075 (TLR7/8, 4 µg/mL), after 1 hour at 37^o^C Brefeldin A (10 µg/mL) and Monensin (10 µg/mL) were added and stimulated cells incubated for a further 3 hours at 37^o^C. After 4 hours of stimulation, each sample was resuspended in 420 µL of PROT1 (Smart Tube Inc) stabilising fixative according to the manufacturer’s instructions. Cryopreserved whole blood samples were stored at –80^o^C. After collection of all samples from each CHMI study, all cryopreserved TLR stimulated whole blood samples were analysed in a single batch. Samples were thawed in an agitating water bath set at room temperature for 10 mins. Thawed samples were transferred into labelled FACS tubes and RBCs lysed according to the manufacturer’s instructions (Smart Tube Inc). In brief, samples were resuspended in 1x Thaw-Lyse buffer, incubated for 10 mins at RT, centrifuged at 600g for 5 minutes at RT. Lysis was repeated, and cell pellets resuspended in 2% FBS/PBS. Resuspended cell pellets were transferred to a 96 V-bottom plate for intracellular cytokine staining. Washed cells were incubated with Fc (1/100, BD Biosciences) and Monocyte Block (1/20, Biolegend) for 10 mins at RT. Cells were resuspended with the surface antibody panel (S6a, S6b Table) and incubated for 15 mins at RT. After washing with 2% FBS/PBS cells were fixed with CytoFix/CytoPerm (BD Biosciences) for 20 mins on ice. After fixation cells were washed with 1 x Permeabilization buffer (BD Biosciences) and resuspended with intracellular antibody mastermix (S6a, S6b Table). Finally, cells were treated with 1x BD Stabilising Fixative (BD Biosciences) for 20 min at RT. Cells were resuspended in 2% FBS/PBS and acquired on a Cytek Aurora 5 within 24 hours.

### Data analysis

#### Flow cytometry.

FACS data was acquired using the Cytek Aurora 5 (CA, USA) and manual gating was performed in FlowJo v10 (BD, 2019). For clustering analysis, we used the R package SPECTRE [67]. Unsupervised clustering was performed and cell clusters were visualized with uniform manifold approximation and projection (UMAP). For functional assays, data was analysed with Flowjo v10 (BD, 2019) and R/R studio was used for statistical analysis and data visualisation.

#### Bulk RNAseq.

Raw sequencing reads were first trimmed to remove adapter sequences and low-quality bases using Cutadapt (v1.9). Trimmed reads were then aligned to the Human GRCh37 reference genome, incorporating Ensembl v97 gene models, using STAR (v2.5.2a). Alignment files were processed, sorted, and converted to the required formats using SAMtools (v1.9). Gene and transcript expression levels were quantified with RSEM (v1.2.30), providing normalized expression estimates. Quality assessment of the RNA-seq data was performed using RNA-SeqQC (v1.1.8) to ensure data reliability. All analyses were carried out using Python (v3.6.1) and Perl (v5.22) for scripting and workflow automation. Low-count genes (fewer than 10 counts) were removed, and dispersion was estimated using edgeR (v4.40) workflow in R. A negative binomial distribution via regression models of normalized count data and Wald test was used to compare gene expression variation between paired pre- and post-stimulated samples from CMV negative and CMV positive with the glmmSeq R package [31]. The design matrix accounted for random effects of individual samples and included an interaction term between state (pre- vs. post-stimulation) and CMV (CMV negative vs. CMV positive). Fold change due to stimulation was calculated by subtracting the log-transformed response term of unstimulated from stimulated samples. Fold change due to CMV was calculated by subtracting the log-transformed response term of CMV negative individuals from CMV positive individuals for both stimulated and unstimulated samples. We corrected for multiple testing using Storey’s q-value method, defining significance for q-values lower than 0.05 with the q-value R package (S2 Table).

### Statistics

Cellular response comparisons within CMV serostatus was performed using non-parametric testing, Wilcoxon signed rank test. For comparisons between CMV groups the Mann Whitney U test was performed. PMR (calculated as multiplication for a 48 hour period) were calculated by applying a sine-wave growth model to the parasitemia data [68]. Speaman’s correlation test was used to assess association between with PMR and cellular responses. P values were not adjusted for multiple comparisons. All statistical analysis were performed in R (version 4.3.2). Graphical outputs were made using the R package ggplot2 (version 3.5.1).

## Supporting information

S1 Table Sampling framework across study. (DOCX)

S2 Table glmmSeq analysis of NK cell response to parasite stimulation in CMV seronegative and positive individuals. (CSV)

S3 Table Demographic characteristics of CHMI cohort. (DOCX)

S4 Table NK cell panel for purity check. (DOCX)

S5 Table NK cell *ex vivo* phenotyping panel in CHMI. (DOCX)

S6 Table S6a/S6b Table: Innate cell whole blood ICS panel. (DOCX)

S1 Fig Transcriptional analysis of NK cells in response to *in vitro* parasite stimulation between CMV seronegative and seropositive individuals. **(A)** Gating example of post isolation QC flowcytometry of NK cells in unstimulated. **(B)** Percentage of live and CD56 cells assessed via flow cytometry after cell-sorting. **(C)** Scatter plots of expression of DEGs grouped based on if they were significant for “CMV”, “State” or “CMV/State Interaction”. Left plots show gene expression at baseline compared to after stimulation with pRBCs and right plots show gene expression levels in CMV seronegative compared to CMV seropositive individuals. **(D)** DEGs identified in glmmSeq that are relatively higher in CMV sero-negative (left panel) and CMV positive (right panel) in unstimulated cells. Related to Fig 1.
(TIF)

S2 Fig NK cells activation in CHMI in CMV seronegative and CMV seropositive individuals. NK cells were analysed during CHMI in CMV seronegative (n = 9) and CMV seropositive individuals (n = 11). (**A)** Gating figure of NK cells in CHMI. **(B)** Proportion of NK subsets at Day 0. **(C)** Total NK cells and its subsets proportions across CHMI. **(D)** Total NK cells and its subsets proportion during malaria in CMV negative and positive separately. **(E)** MFI of activation markers such as Perforin, GrzB, Granulysin, NKp30 and CD38 in CD56bri during malaria. **(F)** MFI of activation markers such as Perforin, GrzB, Granulysin, NKp30 and CD38 in CD56neg during malaria. For **E/F**, data are log_10_ MFIs of markers with thin lines representing individual data coloured by CMV serostatus, and bold lines representing the mean of the predicted values from the fitted models for each group. P values are from linear mixed effect models. CMV is p values for the interaction term between each timepoint (compared to day 0) and CMV serostatus (underlined in grey). NEG/POS are P values for the comparison between day 0 and each subsequent timepoint for CMV seronegative individuals (NEG, underlined in green) and CMV seropositive individuals (POS, underlined in orange) which were determined from contrasts. See also Fig 2. S2 Fig.
(TIF)

S3 Fig Activation and cytotoxicity in NK cell subsets during CHMI. Activation (**A** - CD38 and **B** - NKp30) and cytotoxic (**C** - Perforin, **D** - granulysin, **E** – Granzyme B) were quantified (median fluorescence intensity, MFI) in NK cell subsets that were not otherwise modulated by CMV infection. Expression was compared between day 0 and day 16 in CMV seronegative (n = 9) and CMV seropositive individuals (n = 11), and expression at day 0 or at day 16 was compared between groups. Data are Tukey boxplots with the median, 25^th^ and 75^th^ percentiles. The upper and lower hinges extend to the largest and smallest values, respectively but not further than 1.5X IQR from the hinge. Individual data are shown as points. For comparisons between groups p is Mann-Whitney U test. For comparisons within groups between days p is Wilcoxon signed-rank test.
(TIF)

S4 Fig Cytokine response following TLR stimulation of innate cells. **(A**) Whole blood flow cytometry gating example for identifying classical, non-classical monocytes, DCs and pDCs after stimulation. Total myeloid cells were identified as CD66b/CD3/CD19/CD56 neg HLADR + , classical monocytes were identified as CD14 + , non-classical monocytes (were identified as CD14-/CCR2dim/CD33dim, DCs identified as CD14-/CCR2bright/CD33bright and pDCs as CD123/CD303 + . **(B)** Example flow cytometry plots of cytokine production from total myeloid cells, unstimulated (top line) and stimulated (TLR4) (bottom line).
(TIF)
