# Supplementary material for: Latent cytomegalovirus disrupts innate NK cell responses to P. falciparum and impairs parasite control in first infection in adults
Source: PLoS Pathog. 2026 Jun 23;22(6):e1014372. doi: 10.1371/journal.ppat.1014372 (PMC13309042; doi:10.1371/journal.ppat.1014372)
Supplement: S1 Table — (DOCX) [file ppat.1014372.s001.docx]

Supplementary Table 1: Sampling framework across study

| Assay | Figure | Cohort | CMV status |
| --- | --- | --- | --- |
| Transcriptional response (*in vitro*) | 1A-E, S1 | Malaria naïve healthy donors | Negative n=6  Positive n=6 |
| *Ex vivo* analysis CHMI | 2, 3, 4, 6D, 6E, S3, S4 | Subset of CHMI cohort (see Sup Table 3) | Negative n= 9  Positive n= 11 |
| Myeloid TLR response | 5 | Malaria naïve, healthy donors | Negative n=12  Positive n=17 |
| Clinical associations | 6A, 6B, 6C | See Supplementary Table 3 | Negative n=19  Positive n=21 |
