# Supplementary material for: Latent cytomegalovirus disrupts innate NK cell responses to P. falciparum and impairs parasite control in first infection in adults
Source: PLoS Pathog. 2026 Jun 23;22(6):e1014372. doi: 10.1371/journal.ppat.1014372 (PMC13309042; doi:10.1371/journal.ppat.1014372)
Supplement: S3 Table — (DOCX) [file ppat.1014372.s003.docx]

Supplementary Table 3: Demographic characteristics of CHMI cohort

|  | CMV serostatus | |  |
| --- | --- | --- | --- |
|  | Negative | Positive | P |
| Total, n (%)^ | 19, (48%) | 21, (52%) | 0.751* |
| Sex, male, n (%) | 18 (95%) | 18 (86%) | 1* |
| EBV, positive, n (%) | 18 (95%) | 17 (81%) | 0.865* |
| Age years, median [IQR] | 26 [20.25-31.75] | 25 [21-29] | 0.86# |

* Chi-square, # wilcox rank sum

^ A subset of this cohort were used to investigate NK responses in vivo (Figures 2/3/4)
