# Supplementary material for: Latent cytomegalovirus disrupts innate NK cell responses to P. falciparum and impairs parasite control in first infection in adults
Source: PLoS Pathog. 2026 Jun 23;22(6):e1014372. doi: 10.1371/journal.ppat.1014372 (PMC13309042; doi:10.1371/journal.ppat.1014372)
Supplement: S4 Table — (DOCX) [file ppat.1014372.s004.docx]

Supplementary Table 4: NK cell panel for purity check:

| **Fluorophore** | **Marker** | **Dilution** | **Cat** | **Clone** | **Supplier** | **Lot** |
| --- | --- | --- | --- | --- | --- | --- |
| Live dead aqua | Dead | 1:5000 |  |  |  |  |
| BV421 | CD56 | 1:100 | 562752 | NCAM16.2 | BD | 1110863 |
| BV650 | CD3 | 1:100 | 317324 | OKT3 | Biolegend | B321523 |
| BV785 | HLADR | 1:100 | 307642 | L243 | Biolegend | B324516 |
| FITC | VD1 | 1:50 | TCR2730 | TS8.2 | Invitrogen | UB277210 |
| PerCp-Cy5.5 | CD19 | 1:25 | 561295 | HIB19 | BD | 1159287 |
| PE | CD14 | 1:50 | 561707 | M5E2 | BD | 1349562 |
| PE-Daz | CD64 | 1:100 | 305032 | 10.1 | Biolegend | B292051 |
| APC | VD2 | 1:50 | 331418 | B6 | Biolegend | B352327 |
| AF700 | CD16 | 1:100 | 302026 | 3G8 | Biolegend | B339559 |
