# Supplementary material for: Latent cytomegalovirus disrupts innate NK cell responses to P. falciparum and impairs parasite control in first infection in adults
Source: PLoS Pathog. 2026 Jun 23;22(6):e1014372. doi: 10.1371/journal.ppat.1014372 (PMC13309042; doi:10.1371/journal.ppat.1014372)
Supplement: S5 Table — (DOCX) [file ppat.1014372.s005.docx]

Supplementary Table 5: NK cell *ex vivo* phenotyping panel in CHMI:

| **Fluorophore** | **Marker** | **Dilution** | **Cat** | **Clone** | **Supplier** | **Lot** |
| --- | --- | --- | --- | --- | --- | --- |
| ViadyeRed | Live dead | 1:1000 |  |  | Cytek |  |
| PerCPCy5.5 | TCR γδ | 1:10 | 564157 | B1 | BD | 2207690 |
| BUV661 | LAG3 | 1:25 | 376-2239-42 | 3DS223H | Invitrogen | 2783561 |
| BUV737 | CD56 | 1:100 | 612766 | NCAM16.2 | BD | 1210146 |
| BUV805 | CD3 | 1:400 | 612893 | SK7 | BD | 3212687 |
| BV480 | CD7 | 1:400 | 566119 | MT701 | BD | 2059133 |
| BV570 | HLA-DR | 1:66 | 307638 | I243 | Biolegend | B382459 |
| BV650 | CD14 | 1:200 | 301836 | M5E2 | Biolegend | B360116 |
| BV711 | NKp30 | 1:50 | 563383 | P30-15 | BD | 2131202 |
| BV786 | PD1 | 1:50 | 563789 | EH12.1 | BD | 3276824 |
| BB515 | CD86 | 1:400 | 564545 | 2331 | BD | 9261900 |
| PE | NKG2C | 1:400 | 375004 | S19005E | Biolegend | B370170 |
| PE-CY7 | NKG2A | 1:100 | 375114 | S19004C | Biolegend | B401967 |
| APC | TCR Vδ2 | 1:100 | 331418 | B6 | Biolegend | B352327 |
| efluor660 | CD57 | 1:100 | 50-0577-42 | TB01 | Invitrogen | 2518387 |
| APCfire810 | CD38 | 1:200 | 303550 | HIT2 | Biolegend | B394848 |
| BV421 | Perforin | 1:1600 | 563393 | DG9 | BD | 3254954 |
| AF488 | Granulysin | 1:16 | 558254 | RB1 | BD | 0325603, |
| PE-Fire810 | TIGIT | 1:25 | 372745 | A15153G | Biolegend | B409947 |
| Pe-dazzle594 | CD85j | 1:33 | 333716 | GHI/75 | Biolegend | B375814 |
| AF700 | CD16 | 1:100 | 302026 | 3G8 | Biolegend | B384538 |
| Percp-eFlour710 | CD19 | 1:50 | 46-0198-42 | SJ25C1 | Invitrogen | 2005232 |
| APC | GranzB | 1:1600 | 372204 | QA16A02 | Biolegend | B281725 |
