# Supplementary material for: Latent cytomegalovirus disrupts innate NK cell responses to P. falciparum and impairs parasite control in first infection in adults
Source: PLoS Pathog. 2026 Jun 23;22(6):e1014372. doi: 10.1371/journal.ppat.1014372 (PMC13309042; doi:10.1371/journal.ppat.1014372)
Supplement: S6 Table — (DOCX) [file ppat.1014372.s006.docx]

Supplementary Table 6a: Innate cell whole blood ICS panel

| **Fluorophore** | **Marker** | **Dilution** | **Cat** | **Clone** | **Supplier** | **Lot** |
| --- | --- | --- | --- | --- | --- | --- |
| *Surface stain* | | | | | | |
| BUV496 | CD19 | 1:100 | 12938 | SJ25C1 | BD | 1182272 |
| BUV737 | CD64 | 1:50 | 612776 | 10.1 | BD | 3018874 |
| BUV805 | CD14 | 1:50 | 612902 | M5E2 | BD | 2213039 |
| BV480 | CD86 | 1:50 | 566131 | 2331 | BD | 2136488 |
| BV510 | CD1c | 1:100 | 331534 | L161 | BioLegend | B281571 |
| BV570 | CD33 | 1:50 | 303417 | WM53 | BioLegend | B378792 |
| BV605 | CD303 | 1:50 | 3542274 | 201A | BioLegend | B382651 |
| BV605 | CD123 | 1:100 | 306026 | 6H6 | BioLegend | B379483 |
| BV650 | CD11c | 1:200 | 337238 | Bu15 | BioLegend | B323827 |
| BV750 | CD56 | 1:33 | 362556 | 5.1H11 | BioLegend | B363758 |
| BV785 | HLA-DR | 1:100 | 307642 | L243 | BioLegend | B324516 |
| BB515 | CD3 | 1:100 | 565100 | UCHT1 | BD | 1187749 |
| PerCPCy5.5 | CCR2 | 1:50 | 357204 | K036C2 | BioLegend | B328029 |
| PE-FIRE640 | CD66b | 1:200 | 392918 | 6/40c | BioLegend | B348028 |
| AF700 | CD16 | 1:50 | 302026 | 3G8 | BioLegend | B384538 |
| *Intracellular stain* | | | | | | |
| BUV395 | TNF | 1:25 | 563996 | MAb11 | BD | 1271128 |
| BV421 | IL-12 | 1:25 | 565023 | C8.6 | BD | 2182102 |
| FITC | IL-1b | 1:25 | 11-7013-42 | CRM56 | Thermofisher Invitrogen | 2608896 |
| PE | IL-10 | 1:10 | 559337 | JES3-9D7 | BD | 307185 |
| PE-Cy7 | IL-6 | 1:100 | 501120 | MQ2-13A5 | BioLegend | B320181 |
| AF647 | MCP-1 | 1:50 | 563496 | 5D3-F7 | BD | 9338878 |
| APC | IFNa | 1:5 | 130-092-602 | LT27:295 | Miltenyi Biotec | 5230310707 |

Supplementary Table 6b: Innate cell whole blood ICS panel

| **Fluorophore** | **Marker** | **Dilution** | **Cat** | **Clone** | **Supplier** | **Lot** |
| --- | --- | --- | --- | --- | --- | --- |
| *Surface stain* |  |  |  |  |  |  |
| BUV496 | CD19 | 1:100 | 612938 | SJ25C1 | BD | 1182272 |
| BUV737 | CD64 | 1:50 | 564425 | 10.1 | BD | 8200909 |
| BUV805 | CD14 | 1:50 | 612902 | M5E2 | BD | 1092492 |
| BV480 | CD86 | 1:50 | 566131 | 2331 | BD | 9064680 |
| BB515 | CD3 | 1:100 | 564466 | UCHT1 | BD | 1187749 |
| BV510 | CD56 | 1:100 | 318340 | HCD56 | Biolegend | B367784 |
| BV570 | CD33 | 1:50 | 303417 | WM53 | Biolegend | B332083 |
| BV605 | CD123 | 1:100 | 306026 | 6H6 | Biolegend | B265668 |
| BV650 | CD11c | 1:200 | 117310 | B915 | Biolegend | B323827 |
| BV785 | HLADR | 1:100 | 307642 | L243 | Biolegend | B324516 |
| PerCPCy5.5 | CCR2 | 1:50 | 357204 | K036C2 | Biolegend | B307717 |
| BV605 | CD303 | 1:50 | 354224 | 201A | Biolegend | B349636 |
| PE-FIRE640 | CD66b | 1:100 | 392918 | 6140c | Biolegend | B321127 |
| AF700 | CD16 | 1:100 | 302026 | 3C78 | Biolegend | B333714 |
| APC-FIRE 750 | CD1c | 1:33 | 331545 | L161 | Biolegend | B336699 |
| *Intracellular stain* |  |  |  |  |  |  |
| AF647 | MCP1 | 1:50 | 563496 | 503-F7 | BD | 9338878 |
| BV750 | TNF | 1:5 | 566359 | Mab11 | BD | 1133444 |
| BV421 | IL12 | 1:25 | 565023 | C8-6 | BD | 7263688 |
| PE-Cy7 | IL6 | 1:100 | 501119 | MQ2-13A5 | Biolegend | B281369 |
| FITC | IL1b | 1:25 | 11-7018-42 | CRM56 | Invitrogen | 2527372 |
