# Supplementary material for: Latent cytomegalovirus disrupts innate NK cell responses to P. falciparum and impairs parasite control in first infection in adults
Source: PLoS Pathog. 2026 Jun 23;22(6):e1014372. doi: 10.1371/journal.ppat.1014372 (PMC13309042; doi:10.1371/journal.ppat.1014372)
Supplement: Attachment — Submitted filename: CMV_NK_PlosPathogen_reviewercomments.docx [file ppat.1014372.s012.docx]

We sincerely thank the reviewers for their time in providing helpful feedback on our manuscript and thank the editors for allowing us to revise our manuscript. We have reviewed indicated strengths and weaknesses outlined and have addressed them as described below. Page numbers indicated below are for tracked changes version of our resubmission.

Regarding the following specific focus areas from the editorial comments:

*1) potential new analyses of the impact of the CMV-associated defect on specific NK cell subsets, including the application of specific activation markers noted by reviewers 1 and 2, if those were included in the study;*

Markers IFNγ and CD107a (from R1) were not included in our study as these require stimulation of NK cells, and our analyses of NK cell responses during CHMI was *ex vivo* analysis.

‘Malaria’ topical markers (from R2) were not included in our study as they have been shown to be important in antibody dependent functions of NK cells, while our analysis focuses on innate NK cell responses. Throughout the manuscript we have modified text to emphasis these distinctions.

Unfortunately, samples from the CHMI studies used here have been exhausted and it is therefore not possible to add additional analysis for these markers.

*2) consideration of whether additional functional studies can be applied to bolster the working model that alterations in myeloid derived IL-12 are linked to differential NK cell subset activity/function;*

Due to cell number limitations in each individual study, particularly the CHMI samples, it was not possible to quantify myeloid and NK cell responses within the same individuals. We agree this is a major limitation, and that further mechanistic studies are required. In revision, we have edited text throughout to ensure we do not overstate the data, and to further discuss study limitations.

*3) additional context for the current studies while considering the subsets and markers used here versus those used to subset NK cells in prior work; and*

As outlined below, we have modified text throughout to provide clear context on markers and subsets used here, compared to prior work.

*4) additional clarity on which subjects/samples were included in each experimental analysis.*

We have now stated specifically our experimental approach regarding subjects/samples usage at the beginning of the results and provided a Supplementary Table 1 detailing the breakdown of participants within each figure.

**Part II – Major Issues: Key Experiments Required for Acceptance**

***Reviewer #1:*** *The study omitted some of the most sensitive markers of NK cell activation by pRBC (including CD107a, and CD25 and IFN-gamma, which are particularly relevant for CD56bright cells). It is therefore not appropriate to say that the CMV-induced defect is similar across NK cell subsets.*

Analysis of NK cell responses focused on *ex vivo* analysis of samples from participants during controlled human malaria infection. CD107a and IFNγ were not studied in this assay as those markers require a re-stimulation of PBMCs. We agree with the reviewer that these are important considerations, and these are now mentioned as limitations (line 500). Additionally, we have noted that degranulation was not directly quantified (line 502) and have edited text throughout the manuscript to make clear that cytotoxicity markers are only measuring potential. Further, text has been edited to be more circumstantial regarding whether reduced responses seen in CMV seropositive individuals are a global attenuation.

Despite these limitations, markers used in our *ex vivo* analysis are able to quantify activation in both CD56^dim^ and CD56^bright^ NK cells. Figure 2 shows increased expression of CD38, NKp30, Perforin, Granulysin and GranzymeB during CHMI in CD56^dim^ cells, and corresponding data for CD56^bright^ cells is shown in Supplementary Figure 2D. In both cases, increased marker expression is higher in CMV negative individuals.

*Further, even if the impact of CMV infection were shown to be similar across all subsets, this does not necessarily prove that the defect is NK cell extrinsic. Much more detailed mechanistic studies are required to support such a conclusion.*

*The data do not demonstrate that the observed differences in IL-12 production by myeloid cells are biologically relevant or that such differences would be seen in the context of malaria infection/stimulation. It is thus premature to conclude that reduced IL-12 from myeloid cells explains differences in NK cell activation. Mechanistic, experimental studies are needed to support this conclusion.*

We agree that further mechanistic studies to investigate this mechanism are required. This is now noted in the limitations section (lines 511), and we have edited text throughout the manuscript for clarity regarding potential mechanisms and to ensure we do not overstate conclusions.

**Reviewer #2: Major issues:**
*1. The title and framework of the manuscript don't frame this study from the malaria naive (non-endemic) standpoint clearly. The title makes it sound relevant to all malaria susceptible populations.*

# We have changed to title to reflect the specific relevant populations to “Latent cytomegalovirus disrupts innate NK cell responses to *P. falciparum* and impairs parasite control in first infection in adults”

*CMV seropositivity in the USA and Europe occurs at a lower frequency and much later in life than it does in subsaharan Africa. Most people are positive for CMV between 2-5 years old in Africa and it occurs in teenage years and at less frequency in the subject population used. Whereas, people in subsaharan Africa take years to develop malaria protection after around a decade of already being infected with CMV. Based on this information it wasn't clear if the thrust of this paper was to develop vaccines for malaria naive populations from places where malaria is not endemic or places where it is. The impacts of this research would be very different in the two locations. This needs to be better stated in the introduction and title. To put a different way, the impact of CMV seropositivity and CHMI on a population that is almost 100% positive might be mute as there is no control group. This makes the title a bit over simplified or confusing in scope for the intended impact population or population used.*

As suggested, we have edited the title for clarity. We agree with the reviewer that the early and universal CMV infection in malaria endemic areas is an important consideration in interpreting our data. While the relevance to natural infection is unknown, data highlight the interplay between diverse infecting pathogens on the host immune response, which is of general interest to the immunology and infectious diseases community. We have made edits throughout the manuscript for clarity.

*2. Many phenotypic markers have different outcomes in malaria subjects. NKG2C/CD57 was not seen to correlate to protection in malaria (Hart et al, JEM 2019). Whereas Fc receptor gamma chain negativity seems to be a better marker supported by enhanced functional tests (Hart et al JEM 2019 and Ty et al, Sci Tran Med, 2023). CD56 neg NK cells are in HIV infection have low function, whereas in malaria they seem to be highly functional (FcRg- as well)(Ty et al, Sci Tran Med, 2023). Therefore, the reliance and discussion of many markers that in the past have shown increased functionality I don't think is appropriate considering malaria has been shown to have great effects on cellular function particularly from hemozoin. This does not take away from any effect CMV may have, but it does not allow for any effect of malaria on the system.*

We agree that the impact of prior malaria infection on NK cells and other immune subsets may further modify the impacts of CMV infection reported here. This issue is now included in discussion lines 491-494. Additionally, we have edited the manuscript throughout to clarify our focus on innate NK cell responses.

*3. For the in vitro work, it would be straight forward to first test the proportion of NKG2C+/CD57+ NK cells in the samples used. This would be a potential factor in how much these CMV specific (HLE/CMV peptide) specific NK cells may or may not be effecting the response. Without knowing this proportion, it would be hard to know if they were effecting the response or not. It may be a general phenomena as the authors describe but with lack of a handle on these CMV markers (NKG2C and CD57) and more malaria tropic markers (Fc receptor gamma chain, PLZF, CD56 dim/neg), interpretation of the results throughout is severely limited.*

For *in vitro* analysis of transcriptional responses (Figure 1) NK cells phenotypes were not analyzed prior to stimulation, and we agree this is a limitation. However, evidence of expansion of CMV associated subsets was detected transcriptionally; lines 128-132 describe increased expression of B3GAT1 (CD57), LILRB1 and KLRG2 (NKG2C) in these data.

For ‘malaria’ topical markers, we agree that these may be of further interest, and this is now noted in discussion lines. However, data here focus on innate NK cell responses in naïve individuals. Because these individuals have no prior exposure and therefore no antibodies targeting the parasite, we did not include PLZF and FcRy in our analysis as these markers are involved in antibody dependent functions (Hart JEM 2019). Similarly, while CD56^neg^ subsets expand in areas of high exposure, the role of these cells also appears to be via antibody dependent functions (Ty, STM, 2023). We have revised text throughout to ensure a clear focus on innate NK cell responses to malaria in naïve individuals and during a first malaria infection.

**Reviewer #3: Major issues**

*i) Understandably, it was not possible to perform every PBMC assay on the same individuals, and this is rightly acknowledged as a limitation. My main concern is that this information is not made clear upfront — I only realised much later in the manuscript that the TLR stimulations were not performed on the same individuals as the NK cell analyses. Even now, it is unclear whether every NK cell assay was performed on every participant. The authors could make the paper considerably easier to follow by presenting this information in a table or schematic, and indicating the sample size for each assay and respective groups (as these vary across assays and between groups). If not every NK cell assay was performed on every participant, the authors should discuss how this affects the study's conclusions.*

*Importantly, how were the samples for the different assays selected – and does this bias the results either way?*

We apologies with the confusion regarding sample selection. In revision, we have added an explanation of our analysis approach in the first paragraph of the results (lines 106-111), outlined cohorts in an additional Supplementary Table 1, and clarified sample usage across the cohort throughout results and methods. For NK activation ex vivo, all available samples from the cohort of 40 individuals used in clinical analysis (Figure 6) were used. This information is now included in the methods line 539.

We agree that not analysing all responses in every individual is a limitation, and this is included in limitation lines 506-509.

*ii) Line 200, and repeated elsewhere: the authors state that CMV has a negative effect on malaria-induced activation "across all NK cell subsets." Do they mean all possible NK cell subsets, or all of those specifically studied here? Does this statement contradict line 447?*

Throughout the manuscript we have edited the text for clarity regarding which NK subsets/phenotypes are analysed. For example, line 216 to specify CD56^bright^, CD56^dim^ and CD56^neg^ cells. Limitations line 497 (previously 447) has been expanded to indicate the requirement of future studies with more phenotyping markers and/or unbiased approaches to fully dissect these factors.

*iii) Figure 6: several comparisons contain clear outliers, and the resulting p-values may therefore be misleading (e.g., Figures 6B and 6E). Can the authors comment on the influence of these outliers? This is a particular concern given the small sample sizes.*

All statistical tests use non-parametric approaches. For 6B Mann-Whitney U test was applied and in 6E Spearman’s Rho/p is shown. These non-parametric tests are more robust to outliers thus are unlikely to be unduly influencing findings. Nevertheless, for the reviewer we have analysed Fig 6B without those outliers and included this data here for reviewer confidence. Additionally, within the manuscript clinical outcomes are analysed in two distinct ways – in 6B via clinical score prior to treatment, and then in 6C using ALT as a marker of tissue damage over the course of infection. We agree that our findings require replication in additional and larger studies, and we have now included this in the limitations discussion, line 504.

*iv) The implications of a CMV-driven reduction in parasite control — and potentially in immunopathology, if the outlier-driven result in Figure 6B holds — are substantial for African populations in endemic areas. The authors should suggest how this question might be addressed in these settings, given the high reported prevalence of CMV exposure on the continent.*

We agree that high prevalence and early life acquisition of CMV in malaria endemic areas may make testing the impact of CMV on malaria challenging (or impossible). However, changes to CMV transmission via vaccination or public health measures may open this possibility. In revision we have discussed this further in lines 481-485.

***Part III – Minor Issues: Editorial and Data Presentation Modifications***

***Reviewer #1:*** *The claim that all subsets are similarly affected is in direct contradiction to the statement (in lines 196-202) that defects in activation are more pronounced in CD56br than CD56dim or CD56neg cells? Although, that statement doesn’t really seem justified when looking at the data?*

For clarity, we have revised the wording of this section to more accurately describe the results (lines 210-212).

The comment that these data do not support prior findings – e.g. preferential responsiveness of NKG2A+ NK cells to malaria pRBCs – is inappropriate given that different markers were used to assess activation.

Given the experimental differences we have removed this line from the manuscript (previously line 281).

Similarly, the comment that "NK cell cytotoxicity is associated with PMR" (lines 431-432) is premature - this has not been shown. NK cell cytotoxicity per se was not assessed (just assessment of markers associated with cytotoxic responses) and nor was inhibition of parasite replication by NK cells.

We have edited these lines in the discussion to improve accuracy (lines 421-423), and throughout the manuscript edited for clarity regarding these markers of cytotoxic potential. Additionally, we have noted in limitations specifically that cytotoxicity and parasite killing were not directly assessed (line 502).

EBV serology was performed. What were the results and was there any impact on NK cells?

We have now included EBV seroprevalence in the methods, which was consistently high across all studies, limiting our ability to assess any impact on NK cells or malaria responsiveness. This is now included in limitations lines 514.

***Reviewer #2:*** *(No Response)*

***Reviewer #3:***

*1. Lines 507–508: typo?*

Updated to “and”.

*2. Lines 418–419: Should this be "TLR4 (LPS)"?*

Line edited (now 458).
