# Supplementary material for: Latent cytomegalovirus disrupts innate NK cell responses to P. falciparum and impairs parasite control in first infection in adults
Source: PLoS Pathog. 2026 Jun 23;22(6):e1014372. doi: 10.1371/journal.ppat.1014372 (PMC13309042; doi:10.1371/journal.ppat.1014372)
Supplement: Peer review history

# Peer review: Decision Letter 0

**DOI:** 10.1371/journal.ppat.1014372.r001

**Authors:** Margaret A Phillips, Noah S. Butler

6 May 2026

PPATHOGENS-D-26-00274

Latent cytomegalovirus disrupts NK cell responses to P. falciparum and impairs parasite control

PLOS Pathogens

Dear Dr. Boyle,

Thank you for submitting your manuscript to PLOS Pathogens. After careful consideration, we feel that it has merit but does not fully meet PLOS Pathogens's publication criteria as it currently stands. Therefore, we invite you to submit a revised version of the manuscript that addresses the points raised during the review process.

Please submit your revised manuscript by Jul 05 2026 11:59PM. If you will need more time than this to complete your revisions, please reply to this message or contact the journal office at plospathogens@plos.org. When you're ready to submit your revision, log on to https://www.editorialmanager.com/ppathogens/ and select the 'Submissions Needing Revision' folder to locate your manuscript file.

Please include the following items when submitting your revised manuscript:

* A letter that responds to each point raised by the editor and reviewer(s). You should upload this letter as a separate file labeled 'Response to Reviewers'. This file does not need to include responses to any formatting updates and technical items listed in the 'Journal Requirements' section below.

* A marked-up copy of your manuscript that highlights changes made to the original version. You should upload this as a separate file labeled 'Revised Manuscript with Track Changes'.

* An unmarked version of your revised paper without tracked changes. You should upload this as a separate file labeled 'Manuscript'.

If you would like to make changes to your financial disclosure, competing interests statement, or data availability statement, please make these updates within the submission form at the time of resubmission. Guidelines for resubmitting your figure files are available below the reviewer comments at the end of this letter.

As the corresponding author, your ORCID iD is verified in the submission system and will appear in the published article. PLOS supports the use of ORCID, and we encourage all coauthors to register for an ORCID iD and use it as well. Please encourage your coauthors to verify their ORCID iD within the submission system before final acceptance, as unverified ORCID iDs will not appear in the published article. Only the individual author can complete the verification step; PLOS staff cannot verify ORCID iDs on behalf of authors.

We look forward to receiving your revised manuscript.

Kind regards,

Noah S. Butler

Guest Editor

PLOS Pathogens

Margaret Phillips

Section Editor

PLOS Pathogens

Sumita Bhaduri-McIntosh

Editor-in-Chief

PLOS Pathogens

orcid.org/0000-0003-2946-9497

Michael Malim

Editor-in-Chief

PLOS Pathogens

orcid.org/0000-0002-7699-2064

**Additional Editor Comments:**

There is enthusiasm for the report, however key concerns were raised following review by three experts in the field. Given the significance and power of CHMI studies, the Editors invite the authors to revise the manuscript. Particular attention should be focused on 1) potential new analyses of the impact of the CMV-associated defect on specific NK cell subsets, including the application of specific activation markers noted by reviewers 1 and 2, if those were included in the study; 2) consideration of whether additional functional studies can be applied to bolster the working model that alterations in myeloid derived IL-12 are linked to differential NK cell subset activity/function; 3) additional context for the current studies while considering the subsets and markers used here versus those used to subset NK cells in prior work; and 4) additional clarity on which subjects/samples were included in each experimental analysis.

**Journal Requirements:**

If the reviewer comments include a recommendation to cite specific previously published works, please review and evaluate these publications to determine whether they are relevant and should be cited. There is no requirement to cite these works unless the editor has indicated otherwise.

1) Please ensure that the CRediT author contributions listed for every co-author are completed accurately and in full.

At this stage, the following Authors/Authors require contributions: Reena Mukhiya, Jessica R Loughland, Nicholas L Dooley, Zuleima Pava, Damian Oyong, Dean W Andrew, Julianne Hamelink, Kiana Berry, James S McCarthy, Bridget E Barber, J. Alejandro Lopez, Christian R Engwerda, and Michelle Boyle. Please ensure that the full contributions of each author are acknowledged in the "Add/Edit/Remove Authors" section of our submission form.

The list of CRediT author contributions may be found here: https://journals.plos.org/plospathogens/s/authorship#loc-author-contributions

2) We ask that a manuscript source file is provided at Revision. Please upload your manuscript file as a .doc, .docx, .rtf or .tex. If you are providing a .tex file, please upload it under the item type u2018LaTeX Source Fileu2019 and leave your .pdf version as the item type u2018Manuscriptu2019.

3) Please provide an Author Summary. This should appear in your manuscript between the Abstract (if applicable) and the Introduction, and should be 150-200 words long. The aim should be to make your findings accessible to a wide audience that includes both scientists and non-scientists. Sample summaries can be found on our website under Submission Guidelines:

https://journals.plos.org/plospathogens/s/submission-guidelines#loc-parts-of-a-submission

4) We do not publish any copyright or trademark symbols that usually accompany proprietary names, eg ©,  ®, or TM  (e.g. next to drug or reagent names). Therefore please remove all instances of trademark/copyright symbols throughout the text, including:

- TM on page: 23.

5) Please upload all main figures as separate Figure files in .tif or .eps format. For more information about how to convert and format your figure files please see our guidelines:

https://journals.plos.org/plospathogens/s/figures

6) We have noticed that you have uploaded Supporting Information files, but you have not included a list of legends. Please add a full list of legends for your Supporting Information files after the references list.

7) Please amend your detailed Financial Disclosure statement. This is published with the article. It must therefore be completed in full sentences and contain the exact wording you wish to be published.

1) State what role the funders took in the study. If the funders had no role in your study, please state: "The funders had no role in study design, data collection and analysis, decision to publish, or preparation of the manuscript."

2) If any authors received a salary from any of your funders, please state which authors and which funders..

If you did not receive any funding for this study, please simply state: u201cThe authors received no specific funding for this work.u201d

**Reviewers' Comments:**

Reviewer's Responses to Questions

**Part I - Summary**

Please use this section to discuss strengths/weaknesses of study, novelty/significance, general execution and scholarship.

Reviewer #1: This paper describes a major piece of experimental work to characterize NK cell responses during controlled human malaria infections. The hypothesis is clear and well founded – that persistent, latent CMV infection skews the immune system such that potentially protective responses to malaria parasites are compromised. This hypothesis is underpinned by a considerable body of work describing the impact of CMV infection on many different components of the immune system; numerous studies showing reduced NK cell responsiveness to pathogens, pathogen-associated ligands and vaccines in CMV seropositive individuals; and the authors own work on antibody responses to malaria in CMV infected subjects. Given this existing body of knowledge, the results of this study are very much as expected. The novelty, such as it is, is in the conclusion that the defects are not NK cell intrinsic but rather due to lack of IL-12 from myeloid cells, and that NK cell cytotoxicity is negatively associated with parasite multiplication rate. However, the evidence underpinning each of these conclusions is limited and indirect.

I fully appreciate the difficulty of conducting this type of experiment. The CHMI trials themselves are a major logistic and clinical achievement; adding repeated flow cytometric assessments to the work flow is even more challenging. Conducting a sizable CHMI study simply to explore one hypothesis – such as on NK cells and CMV – is difficult to justify on financial and subject availability grounds such that individual CHMI studies tend to be designed to test a number of hypotheses at the same time. Whilst fully understanding the rationale for this, it does mean that difficult choices have to be made about which cells and cell markers to study at which time point; compromises have to be made. In this case, the compromises seem to be in the choice of NK cell activation markers (some key markers - such as CD25, CD107a and IFN-gamma - were omitted, thereby missing the most sensitive markers of activation of CD56bright NK cells and limiting comparisons with prior studies) and in the choice of stimuli for myeloid cell activation (TLR agonists but nothing malaria specific). These compromises, when combined with the essentially descriptive nature of the study, mean that the novelty of the study derives from rather flimsy observations.

This study definitely deserves to be published but, given the (unavoidable) limitations of the CHMI study design and the lack of any follow-on mechanistic studies to firm up the conclusions, I am not convinced that the data are (yet) sufficiently compelling to warrant publication in PLoS Pathogens.

Reviewer #2: This study looks at malaria-naive individuals that are either seropositive or seronegative for CMV in vitro and correlates outcomes and NK cell phenotype with controlled malaria human infection (CHMI) subject data. The strength of the study is with the unique samples to test their hypothesis. The weaknesses on the manuscript lie in the framing of the study, lack of actual functional data, and lack of inclusion of malaria specific NK markers shown to correlate with protection.

Reviewer #3: This manuscript presents additional analyses of PBMCs from a series of induced P. falciparum blood-stage challenges in malaria-naïve Australian volunteers — a resource that has already proved highly informative for malaria immunology. The current analysis examines the effect of latent CMV seropositivity on immune responses to P. falciparum. Given the established consensus that CMV negatively affects immune responses to other pathogens, including those induced by vaccination, this is an important question for malaria, which remains an intractable public health problem in Africa and parts of Asia. The authors report reduced responsiveness of NK cells in CMV seropositive compared with seronegative individuals, reduced control of parasite growth, and an initial investigation of the mechanisms underlying this suppression.

The manuscript is a valuable addition to the immunology literature and, on the whole, is clearly written. The data have been carefully parsed and tell a coherent story.

**********

**Part II – Major Issues: Key Experiments Required for Acceptance**

Please use this section to detail the key new experiments or modifications of existing experiments that should be absolutely required to validate study conclusions.

Generally, there should be no more than 3 such required experiments or major modifications for a "Major Revision" recommendation. If more than 3 experiments are necessary to validate the study conclusions, then you are encouraged to recommend "Reject".

Reviewer #1: The study omitted some of the most sensitive markers of NK cell activation by pRBC (including CD107a, and CD25 and IFN-gamma, which are particularly relevant for CD56bright cells). It is therefore not appropriate to say that the CMV-induced defect is similar across NK cell subsets.

Further, even if the impact of CMV infection were shown to be similar across all subsets, this does not necessarily prove that the defect is NK cell extrinsic. Much more detaiied mechanistic studies are required to support such a conclusion.

The data do not demonstrate that the observed differences in IL-12 production by myeloid cells are biologically relevant or that such differences would be seen in the context of malaria infection/stimulation. It is thus premature to conclude that reduced IL-12 from myeloid cells explains differences in NK cell activation. Mechanistic, experimental studies are needed to support this conclusion.

Reviewer #2: Major issues:

1. The title and framework of the manuscript don't frame this study from the malaria naive (non-endemic) standpoint clearly. The title makes it sound relevant to all malaria susceptible populations. CMV seropositivity in the USA and Europe occurs at a lower frequency and much later in life than it does in subsaharan Africa. Most people are positive for CMV between 2-5 years old in Africa and it occurs in teenage years and at less frequency in the subject population used. Whereas, people in subsaharan Africa take years to develop malaria protection after around a decade of already being infected with CMV. Based on this information it wasn't clear if the thrust of this paper was to develop vaccines for malaria naive populations from places where malaria is not endemic or places where it is. The impacts of this research would be very different in the two locations. This needs to be better stated in the introduction and title. To put a different way, the impact of CMV seropositivity and CHMI on a population that is almost 100% positive might be mute as there is no control group. This makes the title a bit over simplified or confusing in scope for the intended impact population or population used.

2. Many phenotypic markers have different outcomes in malaria subjects. NKG2C/CD57 was not seen to correlate to protection in malaria (Hart et al, JEM 2019). Whereas Fc receptor gamma chain negativity seems to be a better marker supported by enhanced functional tests (Hart et al JEM 2019 and Ty et al, Sci Tran Med, 2023). CD56 neg NK cells are in HIV infection have low function, whereas in malaria they seem to be highly functional (FcRg- as well)(Ty et al, Sci Tran Med, 2023). Therefore, the reliance and discussion of many markers that in the past have shown increased functionality I don't think is appropriate considering malaria has been shown to have great effects on cellular function particularly from hemozoin. This does not take away from any effect CMV may have, but it does not allow for any effect of malaria on the system.

3. For the in vitro work, it would be straight forward to first test the proportion of NKG2C+/CD57+ NK cells in the samples used. This would be a potential factor in how much these CMV specific (HLE/CMV peptide) specific NK cells may or may not be effecting the response. Without knowing this proportion, it would be hard to know if they were effecting the response or not. It may be a general phenomena as the authors describe but with lack of a handle on these CMV markers (NKG2C and CD57) and more malaria tropic markers (Fc receptor gamma chain, PLZF, CD56 dim/neg), interpretation of the results throughout is severely limited.

Reviewer #3: i) Understandably, it was not possible to perform every PBMC assay on the same individuals, and this is rightly acknowledged as a limitation. My main concern is that this information is not made clear upfront — I only realised much later in the manuscript that the TLR stimulations were not performed on the same individuals as the NK cell analyses. Even now, it is unclear whether every NK cell assay was performed on every participant. The authors could make the paper considerably easier to follow by presenting this information in a table or schematic, and indicating the sample size for each assay and respective groups (as these vary across assays and between groups). If not every NK cell assay was performed on every participant, the authors should discuss how this affects the study's conclusions. Importantly, how were the samples for the different assays selected – and does this bias the results either way?

ii) Line 200, and repeated elsewhere: the authors state that CMV has a negative effect on malaria-induced activation "across all NK cell subsets." Do they mean all possible NK cell subsets, or all of those specifically studied here? Does this statement contradict line 447?

iii) Figure 6: several comparisons contain clear outliers, and the resulting p-values may therefore be misleading (e.g., Figures 6B and 6E). Can the authors comment on the influence of these outliers? This is a particular concern given the small sample sizes.

iv) The implications of a CMV-driven reduction in parasite control — and potentially in immunopathology, if the outlier-driven result in Figure 6B holds — are substantial for African populations in endemic areas. The authors should suggest how this question might be addressed in these settings, given the high reported prevalence of CMV exposure on the continent.

**********

**Part III – Minor Issues: Editorial and Data Presentation Modifications**

Please use this section for editorial suggestions as well as relatively minor modifications of existing data that would enhance clarity.

Reviewer #1: The claim that all subsets are similarly affected is in direct contradiction to the statement (in lines 196-202) that defects in activation are more pronounced in CD56br than CD56dim or CD56neg cells? Although, that statement doesn’t really seem justified when looking at the data?

The comment that these data do not support prior findings – e.g. preferential responsiveness of NKG2A+ NK cells to malaria pRBCs – is inappropriate given that different markers were used to assess activation. .

Similarly, the comment that "NK cell cytotoxicity is associated with PMR" (lines 431-432) is premature - this has not been shown. NK cell cytotoxicity per se was not assessed (just assessment of markers associated with cytotoxic responses) and nor was inhibition of parasite replication by NK cells.

EBV serology was performed. What were the results and was there any impact on NK cells?

Reviewer #2: (No Response)

Reviewer #3: 1. Lines 507–508: typo?

2. Lines 418–419: Should this be "TLR4 (LPS)"?

**********

PLOS authors have the option to publish the peer review history of their article (what does this mean?). If published, this will include your full peer review and any attached files.

If you choose “no”, your identity will remain anonymous but your review may still be made public.

**Do you want your identity to be public for this peer review?**    For information about this choice, including consent withdrawal, please see our Privacy Policy.

Reviewer #1: No

Reviewer #2: No

Reviewer #3: No

**Figure resubmission:**

While revising your submission, we strongly recommend that you use PLOS’s NAAS tool (https://ngplosjournals.pagemajik.ai/artanalysis) to test your figure files. NAAS can convert your figure files to the TIFF file type and meet basic requirements (such as print size, resolution), or provide you with a report on issues that do not meet our requirements and that NAAS cannot fix.

After uploading your figures to PLOS’s NAAS tool - https://ngplosjournals.pagemajik.ai/artanalysis, NAAS will process the files provided and display the results in the "Uploaded Files" section of the page as the processing is complete. If the uploaded figures meet our requirements (or NAAS is able to fix the files to meet our requirements), the figure will be marked as "fixed" above. If NAAS is unable to fix the files, a red "failed" label will appear above. When NAAS has confirmed that the figure files meet our requirements, please download the file via the download option, and include these NAAS processed figure files when submitting your revised manuscript.

**Reproducibility:**

To enhance the reproducibility of your results, we recommend that authors of applicable studies deposit laboratory protocols in protocols.io, where a protocol can be assigned its own identifier (DOI) such that it can be cited independently in the future. Additionally, PLOS ONE offers an option to publish peer-reviewed clinical study protocols. Read more information on sharing protocols at https://plos.org/protocols?utm_medium=editorial-email&utm_source=authorletters&utm_campaign=protocols

# Author comment: Author response to Decision Letter 1

**DOI:** 10.1371/journal.ppat.1014372.r002

2 Jun 2026

Attachment Submitted filename: CMV_NK_PlosPathogen_reviewercomments.docx

# Editor report: Decision Letter 1

**DOI:** 10.1371/journal.ppat.1014372.r003

**Authors:** Margaret A Phillips, Noah S. Butler

11 Jun 2026

Dear Dr. Boyle,

We are pleased to inform you that your manuscript 'Latent cytomegalovirus disrupts innate NK cell responses to P. falciparum and impairs parasite control in first infection in adults' has been provisionally accepted for publication in PLOS Pathogens.

Before your manuscript can be formally accepted you will need to complete some formatting changes, which you will receive in a follow up email. A member of our team will be in touch with a set of requests.

Please note that your manuscript will not be scheduled for publication until you have made the required changes, so a swift response is appreciated.

IMPORTANT: The editorial review process is now complete. PLOS will only permit corrections to spelling, formatting or significant scientific errors from this point onwards. Requests for major changes, or any which affect the scientific understanding of your work, will cause delays to the publication date of your manuscript.

Should you, your institution's press office or the journal office choose to press release your paper, you will automatically be opted out of early publication. We ask that you notify us now if you or your institution is planning to press release the article. All press must be co-ordinated with PLOS.

Thank you again for supporting Open Access publishing; we are looking forward to publishing your work in PLOS Pathogens.

Best regards,

Noah S. Butler

Guest Editor

PLOS Pathogens

Margaret Phillips

Section Editor

PLOS Pathogens

Sumita Bhaduri-McIntosh

Editor-in-Chief

PLOS Pathogens

orcid.org/0000-0003-2946-9497

Michael Malim

Editor-in-Chief

PLOS Pathogens

orcid.org/0000-0002-7699-2064

***********************************************************

The authors have carefully and revised the manuscript and provided thoughful responses to the reviewer queries.

Reviewer Comments (if any, and for reference):

# Editor report: Acceptance letter

**DOI:** 10.1371/journal.ppat.1014372.r004

**Authors:** Margaret A Phillips, Noah S. Butler

Dear Dr. Boyle,

We are delighted to inform you that your manuscript, "

Latent cytomegalovirus disrupts innate NK cell responses to P. falciparum and impairs parasite control in first infection in adults," has been formally accepted for publication in PLOS Pathogens.

We have now passed your article onto the PLOS Production Department who will complete the rest of the pre-publication process. All authors will receive a confirmation email upon publication.

The corresponding author will soon be receiving a typeset proof for review, to ensure errors have not been introduced during production. Please review the PDF proof of your manuscript carefully, as this is the last chance to correct any scientific or type-setting errors. Please note that major changes, or those which affect the scientific understanding of the work, will likely cause delays to the publication date of your manuscript. Note: Proofs for Front Matter articles (Pearls, Reviews, Opinions, etc...) are generated on a different schedule and may not be made available as quickly.

Soon after your final files are uploaded, the early version of your manuscript, if you opted to have an early version of your article, will be published online. The date of the early version will be your article's publication date. The final article will be published to the same URL, and all versions of the paper will be accessible to readers.

For Research Articles, you will receive an invoice from PLOS for your publication fee after your manuscript has reached the completed accept phase. If you receive an email requesting payment before acceptance or for any other service, this may be a phishing scheme. Learn how to identify phishing emails and protect your accounts at https://explore.plos.org/phishing.

Thank you again for supporting open-access publishing; we are looking forward to publishing your work in PLOS Pathogens.

Best regards,

Sumita Bhaduri-McIntosh

Editor-in-Chief

PLOS Pathogens

orcid.org/0000-0003-2946-9497

Michael Malim

Editor-in-Chief

PLOS Pathogens

orcid.org/0000-0002-7699-2064
